# Antimicrobial Lipids from Plants and Marine Organisms: An Overview of the Current State-of-the-Art and Future Prospects

**DOI:** 10.3390/antibiotics9080441

**Published:** 2020-07-24

**Authors:** Eliana Alves, Marina Dias, Diana Lopes, Adelaide Almeida, Maria do Rosário Domingues, Felisa Rey

**Affiliations:** 1 Mass Spectrometry Centre, LAQV-REQUIMTE, Department of Chemistry, University of Aveiro, Campus Universitário de Santiago, 3810-193 Aveiro, Portugal; marinadias@ua.pt (M.D.); dianasalzedaslopes@ua.pt (D.L.); mrd@ua.pt (M.d.R.D.); 2 Centre for Environmental and Marine Studies, CESAM, Department of Chemistry, University of Aveiro, Campus Universitário de Santiago, 3810-193 Aveiro, Portugal; 3 Centre for Environmental and Marine Studies, CESAM, Department of Biology, University of Aveiro, Campus Universitário de Santiago, 3810-193 Aveiro, Portugal; aalmeida@ua.pt

**Keywords:** fatty acid, lipid extract, lipidomics, macroalga, marine invertebrate, mechanism of action, microalga, minimum inhibitory concentration, monoacylglycerol, natural antimicrobial

## Abstract

In the actual post-antibiotic era, novel ways of rethinking antimicrobial research approaches are more urgent than ever. Natural compounds with antimicrobial activity such as fatty acids and monoacylglycerols have been investigated for decades. Additionally, the interest in other lipid classes as antimicrobial agents is rising. This review provides an overview on the research about plant and marine lipids with potential antimicrobial activity, the methods for obtaining and analyzing these compounds, with emphasis on lipidomics, and future perspectives for bioprospection and applications for antimicrobial lipids. Lipid extracts or lipids isolated from higher plants, algae or marine invertebrates are promising molecules to inactivate a wide spectrum of microorganisms. These lipids include a variety of chemical structures. Present and future challenges in the research of antimicrobial lipids from natural origin are related to the investment and optimization of the analytical workflow based on lipidomics tools, complementary to the bioassay-guided fractionation, to identify the active compound(s). Also, further work is needed regarding the study of their mechanism of action, the structure–activity relationship, the synergistic effect with conventional antibiotics, and the eventual development of resistance to lipids, which, as far as is known, is unlikely.

## 1. Introduction

The consumption of antibiotics in the world population is alarming. In 2016, the top five World Health Organization (WHO)’s major antibiotic consuming countries were Brazil, Turkey, Iran, Russia, and France, by decreasing order [1]. In Brazil, more than 2000 metric tons of antibiotics were consumed annually, followed by ca. 1000 metric tons in Turkey and Iran [1].

Both misuse and overuse of antibiotics has led to the development of antimicrobial resistance (AMR) in microorganisms, which has been a global problem and a growing threat for many years. Antimicrobial resistant microbes are found in people, animals, food, and the environment (hospital or other health care facilities, water, soil and air). Because of AMR, several disease conditions are becoming harder to treat, as tuberculosis, pneumonia, blood poisoning, gonorrhea, and foodborne diseases [2]. AMR leads to higher medical costs, prolonged hospital stays, and increased mortality, causing an economic burden for health care systems. The major cause of AMR is mostly due to misuse of antibiotics.

A number of 700,000 deaths occur worldwide because of drug-resistant diseases [3]. Tuberculosis causes 1.8 million deaths per year, while multidrug-resistant (MDR) tuberculosis causes 250,000 deaths per year and is a global priority for research and development. Gram-negative bacteria can cause death in days because of the lack of treatment options. By 2050, it is foreseen that drug-resistant diseases could cause 10 million deaths each year [3]. In 2017, the WHO identified a priority list of highly antimicrobial-resistant pathogenic microorganisms, also known as superbugs, that have developed survival mechanisms to circumvent the action of last-line antimicrobials (isoniazid, rifampicin, fluoroquinolone, carbapenem, third-generation cephalosporin, or vancomycin) [4]. There are twelve bacteria that have critical and high priority for treatment discovery, besides *Mycobacterium tuberculosis*, the causing agent of tuberculosis, including the “ESKAPE” pathogens: *Enterococcus faecium*, *Staphylococcus aureus*, *Klebsiella pneumoniae*, *Acinetobacter baumannii*, *Pseudomonas aeruginosa*, and Enterobacteriaceae [5]. Superbugs cause 33,000 deaths each year, in Europe, by antibiotic-resistant bacterial infections. Italy is the European country with one-third of all cases (11,000 deaths in total), followed by France (more than 5500 deaths) and Germany (with 2300 deaths) [6,7].

The world now lives the so-called “post-antibiotic era.” The current guidelines and recommendations from the WHO claim for an interconnected action and national action plans in a multisectoral and sustained “One Health” approach. This is aimed to tackle AMR and achieve the United Nations’ Sustainable Development Goals for 2030 toward humans, food and feed, plants and crops, environment, terrestrial and aquatic animals [8].

According to a recent WHO’s report, there are 252 antimicrobial agents in preclinical pipeline, being developed to treat WHO’s priority pathogens, but at very early stages of development [9]. Even so, very few target the most critical resistant Gram-negative bacteria, thus, they will generate little benefit over existing treatments [9]. As such, it appears that the future will come up with an increased need for new compounds with antimicrobial activity and combined therapeutic strategies, which can be effective against superbugs and bring revenue to the pharmaceutical industry. At the same time, several alternative approaches to conventional antibiotics have been extensively studied, not only to be used in the clinical field but also in animal health, control insect pest, protect agricultural crops, improve food safety, and water disinfection. Developing strategies include antimicrobial peptides (AMP), phage therapy, photodynamic antimicrobial chemotherapy (PACT), nanoparticles, probiotics, lysins, antibodies, quorum sensing inhibitors, and immunotherapeutic agents [5,10,11,12,13,14]. Combination therapy or multi-target approaches are being developed to hinder antibiotic resistance or to sensitize microorganisms to antibiotic action [15]. Another strategy to overcome AMR is the combination of conventional antibiotics with other molecules, as natural products and/or antimicrobials from natural sources, as plants and marine organisms, to enhance the antimicrobial effect against a wide range of pathogens.

Medicinal plants and marine organisms are natural sources of many antimicrobial compounds [14,16,17,18]. Plant components with antimicrobial activity include alkaloids, sulfur-containing compounds, diterpenes/terpenoids [19], fatty acids (FA) [20,21,22], some carbohydrates [23], steroidal glycosides, and phenolic compounds [24]. Both primary and secondary metabolites are “generally recognized as safe” (GRAS) substances and the chance of triggering antimicrobial resistance is low [25]. Simultaneously, marine organisms, mainly slow-moving or sessile, have developed adaptive defense mechanisms to protect themselves against pathogenic microorganisms. In some cases, marine organisms maintain associations with microbiota, being bacterial symbionts responsible by the synthesis of antimicrobial molecules [26,27].

### 1.1. Synergistic Effects between Natural Products and Antibiotics

Phytochemicals exert potential antimicrobial activities against both resistant and sensitive pathogenic microorganisms through distinct mechanisms of action. Additionally, some of them exert in vitro synergistic effects when combined with conventional antibiotics [19]. These natural products have intrinsic antibacterial, antiviral, antifungal, and antiparasitic activity, usually in higher concentrations than synthetic compounds, but they can be used to potentiate the effect of drugs. Synergistic effects have been observed by simultaneous administration of phytochemicals with antibiotics by increasing the effectiveness of oxacillin, tetracycline, nalidixic acid, ofloxacin, chloramphenicol, gentamicin, erythromycin, penicillin, ampicillin, kanamycin, and ciprofloxacin, primarily antibiotics from the group of inhibitors of cell wall synthesis and protein synthesis [25,28]. Likewise, the combination of phytochemical with antibiotics significantly reduced the values of their minimum inhibitory concentrations (MIC) [19].

On the other side, the effects of antimicrobial compounds from marine organisms in combination with conventional antibiotics have been poorly explored. Synergistic interactions were found in combination of long chain polyunsaturated FA (PUFA) with benzoyl peroxide to inhibit the growth of *S. aureus* [29]. This synergistic effect of FA suggests an increase in the cell membrane’s permeability, improving the penetration of the antimicrobial agent [29]. Fucoidan increased oral bacterial killing when combined with antibiotics [30]. Two AMP, tachycitin and a big defensin from the horseshoe crab *Tachypleus tridentatus* acted synergistically in antimicrobial activities [31]. Fucofuroeckol-A from the kelp *Eisenia bicyclis* reversed erythromycin and lincomycin resistance of *Propionibacterium acnes* and demonstrated a weak synergistic effect with both antibiotics [32]. Two marine oligosaccharides showed a synergistic effect with azithromycin against a wild-type strain of *P. aeruginosa* [33].

### 1.2. Antimicrobial Lipids

FA, monoacylglycerols (MAG), sterols and terpene derivatives have been the most studied antimicrobial lipid classes over the decades [20,21,22,34,35]. The efficiency of these lipids over a wide spectrum of microorganisms is linked to their chemical structure and is influenced by the pH of the medium. The structure-activity relationship between free FA (non-esterified) and bacteria is based on the chemical structure of the FA and depends on the acyl chain length, the stereochemistry, the degree of unsaturation, or on the esterified form of FA [20,21]. Short-chain FA (C6 or lower) are effective against Gram-negative bacteria (high concentration and pH-dependent); long-chain FA (C12 or higher) are effective against Gram-positive bacteria (low concentration and pH-dependent); methyl esters of FA (FAME) decrease bacterial activity; sucrose esters increase bacterial activity; *cis*-isomers are more active than *trans*-isomers; unsaturated ones increase the activity against Gram-negative bacteria [21]. FA and MAG are mostly effective against Gram-positive bacteria, quite possibly because of the complexity of the cell wall of Gram-negative bacteria, and the lower complexity and greater porosity of the Gram-positive bacterial wall, made up of several layers of peptidoglycan [36], which provides a degree of porosity to bacteria that allows the penetration of the antimicrobial agent into the cell [10]. In these bacteria, the absence of an outer membrane and the porous structure of the peptidoglycan cell wall allow the entry of antimicrobial molecules through the cell wall [37]. FA are amphipathic molecules with the hydrocarbon chain representing the hydrophobic part, while the carboxylic acid group is hydrophilic. This group can be polar or anionic in aqueous solution depending on the pH of the medium. However, antimicrobial inactivation studies with free FA isolated from biological matrices (plants or marine organisms) deserve greater attention in the near future, since FA with antimicrobial properties are sometimes identified as the most abundant compounds in the active extracts. However, when they are isolated, they do not exert any activity against the tested microorganisms.

FA have also shown antifungal activity and have the advantage of being non-toxic to the environment as conventional antifungals. Although they have a high degree of specificity, the probability of developing resistance in pathogenic fungi is low [35]. The most important target of antifungal FA is the cell membrane. They increase the fluidity of the membrane, leading to leakage of intracellular components and cell death. Other targets include protein synthesis that can be inhibited by myristic acid (14:0) analogues, FA metabolism and topoisomerase activity that can be inhibited by acetylenic FA, among others [35]. Some are FA produced by plants as a defense mechanism against fungal attack such as acetylenic FA. These FA have a triple bond (C≡C) and their putative mechanism of action is the inhibition of sphingolipid synthesis [38]. Fungi are more inhibited by acetylenic FA derivatives than by ethylenic FA derivatives (FA with double bonds) [21]. Cyclopropane FA, which have a cyclic structure, also revealed antifungal activity [35]. The synergism of antifungal drugs with antifungal FA increases the susceptibility to those drugs, potentiating and prolonging their effect [39].

The antifungal efficiency of free FA increases with the increase in the carbon chain length. FA with proven activity against several fungi, both yeast and mold species, include saturated (4:0 to 16:0) and unsaturated (4:1 to 22:5) ones, which increase the fungicidal activity because of their greater freedom of movement within the cell membrane [40]. Methylated FA, also known as branched FA, (6-Me 17:1 and 12-Me 14:0), several saturated and unsaturated oxylipins (12:0 to 18:3) having one to three hydroxyl (OH-) groups have shown antifungal activity as well [40]. Oxylipins are originated from oxidation of PUFA and include different types of oxygenated FA derivatives.

A strong synergistic effect was evidenced on the antimicrobial action of linoleic acid (18:2) and oleic acid (18:1) against *S. aureus* and *Kocuria kristinae,* formerly known as *Micrococcus kristinae*, and also, a strong synergistic effect between 18:2 and MAG [monolaurin, MAG(C12:0)], or monomyristin, [MAG(C14:0)] greater than the isolated effect of 18:2 [41]. The 10- and 14-carbon MAG were effective against *Helicobacter pylori*, but free FA 14:1 and linolenic acid (18:3) were the most effective against this bacterium [42]. MAG(C10:0) was effective against *Neisseria ghonorroeae*, MAG(C12:0) against *S. aureus* and FA 12:0 against methicillin-resistant *S. aureus* (MRSA) and methicillin-sensitive *S. aureus* (MSSA), as recently reviewed by Yoon et al. (2018) [21]. A great advantage of antimicrobial lipids is that they are difficult to trigger resistance mechanisms in microbes, as observed for pathogenic fungi, as mentioned above [35] and also for bacteria, since they can grow in culture medium in the presence of these lipids, in sublethal concentrations, for up to a year without developing resistance [43].

Despite the promising results, mostly performed with pure commercial compounds, it is necessary to get an insight on the bioactive lipid molecules from natural sources. In some cases, pure lipid compounds were obtained from plant or marine organism extracts after several fractionation and purification steps, for further analysis and identification. There are few research works that were able to isolate a single lipid compound or a class of lipids. Additionally, a complex task is to verify the structure-activity relationship that requires further studies. Electron microscopy techniques and model membrane systems are useful tools to understand the mode of action of lipids toward the microorganisms. This laborious work requires a concerted action from several scientific disciplines as well as many methodological and technological fields and specialized personnel.

Thus, the study of lipids as molecules with antimicrobial potential, may be enhanced in the short term, with the help of lipidomics as an emerging tool for the identification and characterization of lipids (Section 5). This will promote the accurate identification of bioactive compounds, the discovery of new lipids and, eventually, compounds that can be used in combination with existing drugs to aid therapy.

**Figure 1:**
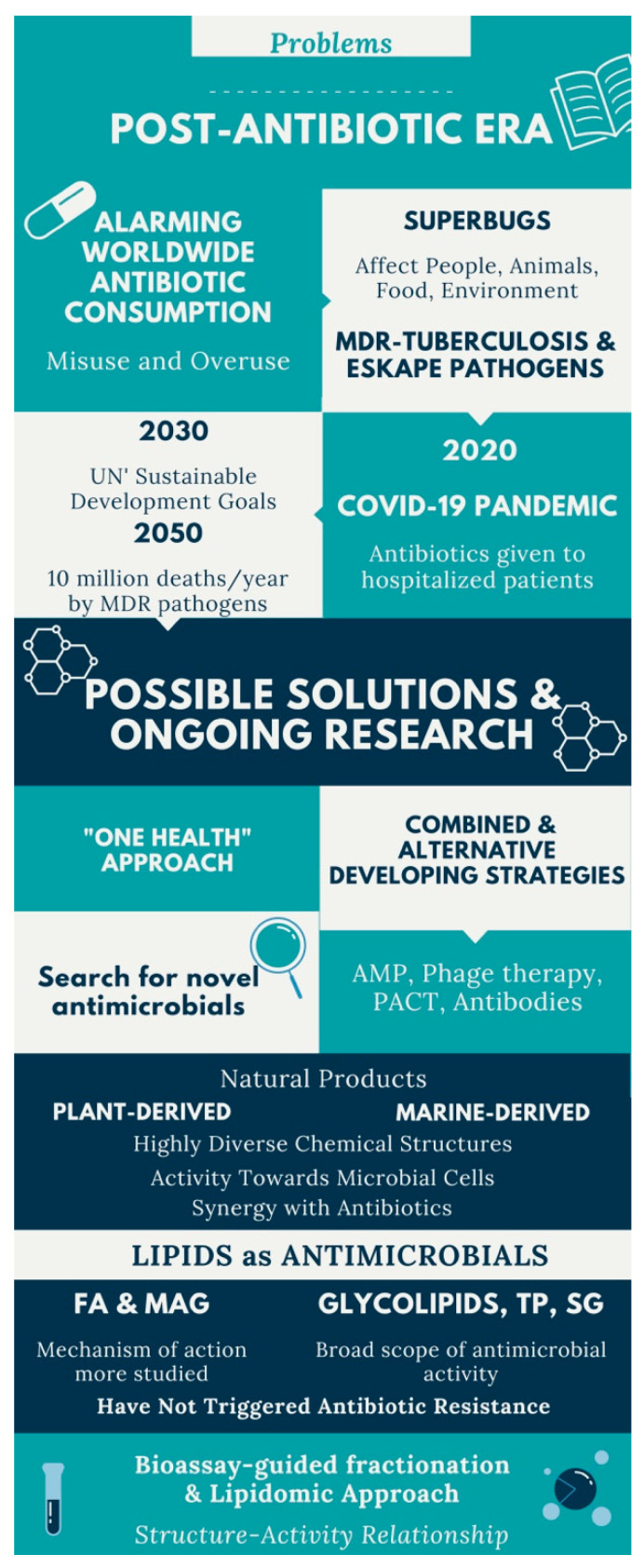
Infographic of the actual state-of-the-art on antibiotic resistance and search for novel antimicrobials, as natural product-derived lipids. Abbreviations: MDR, multidrug resistant; UN, United Nations; AMP, antimicrobial peptides; PACT, photodynamic antimicrobial chemotherapy; FA, fatty acids; MAG, monoacylglycerols; TP, terpenoids; SG, steroidal glycosides.

A summary of the actual state-of-the-art on antibiotic resistance and search for novel antimicrobials, as natural product-derived lipids, is illustrated in Figure 1.

### 1.3. Aim of the Study

This review aims to provide a critical overview on the research about plant and marine lipids with potential antimicrobial activity, the methods for obtaining and analyzing these compounds with emphasis on lipidomics, and future perspectives for bioprospecting and applying these antimicrobial lipids.

## 2. Antimicrobial Lipids from Plants

Over the evolution, higher plants have developed several resilience strategies that allow them to resist or escape external attacks (e.g., microorganisms, pathogens, and predators). Their innate immune system had to be equipped with highly complex mechanisms of resistance and survival. The defense mechanisms of plants are unique and consist of both physical barriers and production of secondary metabolites. Plant secondary metabolites are formed in particular biosynthetic pathways by means of substrate-specific enzymes. The precursors of these secondary metabolites stem from primary metabolites, such as amino acids, FA, sugars, or acetyl-CoA. Some of the secondary metabolites serve as constitutive chemical barriers against the microbial attack (phytoanticipins) while others serve as inducible antimicrobials (phytoalexins) [44].

Oxylipins are a large family of plants’ secondary metabolites derived from PUFA that make part of their immune system and play key roles as antimicrobial agents. They are formed through enzymatic or radical oxidation of FA 18:2 and 18:3, in order to protect plants against pests and pathogens [45]. The enzymatic biosynthesis of these molecules is triggered by an alpha-dioxygenase (DOX) and by lipoxygenases (LOX) [46,47] that lead to the formation of the different types of molecules, including hydroxy-, hydroperoxy-, divinyl-, oxo-, and keto-derivatives of FA. Oxylipins are formed during abiotic and biotic stresses [48]. They are plant signaling molecules that can induce cell death and have an effect on the growth of eukaryotic microbes [45]. A deeper knowledge on plant response to stresses at molecular, physiological and metabolic levels will allow the development of new plant-derived antimicrobial molecules for use in the clinical field and as biopesticides [48].

The search for novel antimicrobials has led to exploring also amide derivatives of FA because they are natural self-defense agents in plants. FA amides are bioactive lipids [49] formed by the amidation of long chain saturated and unsaturated FA (UFA) [50]. They have higher antimicrobial activity against yeasts and bacteria than unmodified FA [51]. Amide derivatives of FA possess a broad bioactivity against different pathologic conditions (bacterial and parasitic infections, cancer, inflammation, etc.,) and their mechanisms of action imply protein synthesis inhibition and membrane leakage [52]. Also, microorganisms inside healthy plant tissues are unique to explore novel bioactive compounds. The FA and their amides from plant’s endogenous microorganisms have been scarcely reported despite being bioactive in a variety of processes and should be more explored as new therapeutic agents [52].

**Figure 2:**
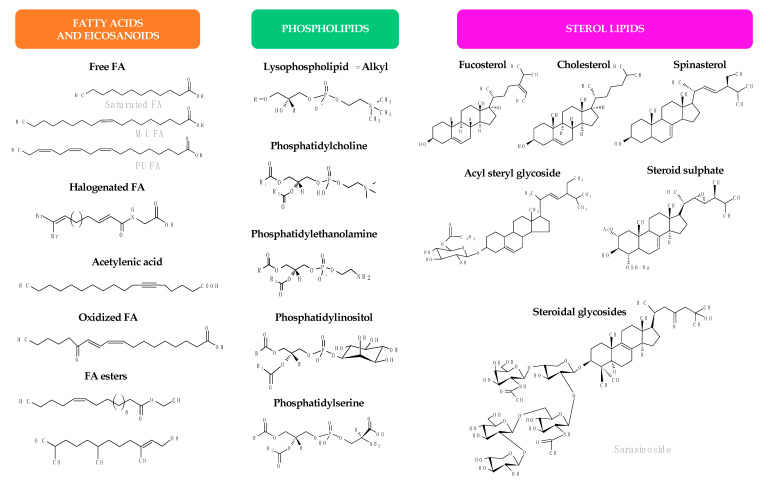

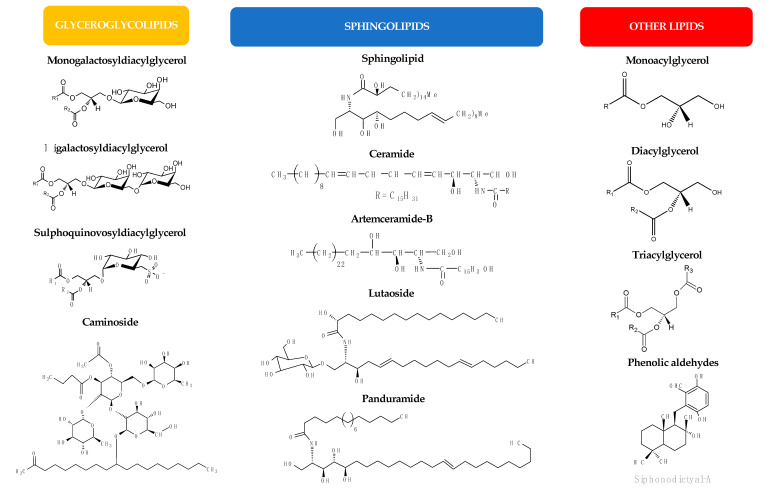

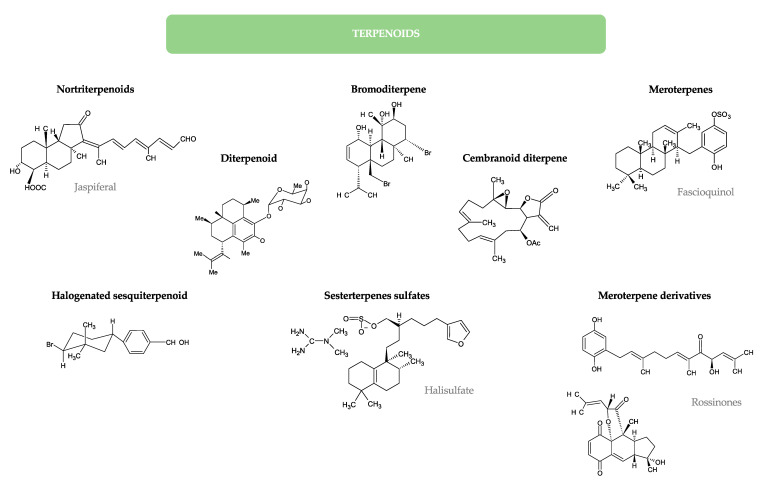
Chemical structures of the different lipid classes isolated from natural sources with antimicrobial activity.

Lipids represent up to 7% of the dry weight of the leaves of higher plants and are important constituents of cell membranes, chloroplasts, and mitochondria [53]. Besides their structural function as main constituents of cell membranes, they have functional roles in plants (intracellular mediators, extracellular signalers, inter-species communication, and plant defense) and also serve as energy reserves (namely in seeds during germination) [21]. Palmitic acid (16:0) is the major saturated FA in leaf lipids. On the other hand, chloroplast membranes can have up to 90% α-18:3 FA in some lamellae [21]. FA exist in plants mainly linked to more complex molecules, as acylglycerols, esterified to a glycerol backbone in the form of triacylglycerols, sterol esters, MAG and diacylglycerols, phospholipids, or glycolipids. Several lipid classes, besides FA and MAG, have been identified in a diverse group of higher plants and tested for their antimicrobial activity, as will be detailed in the next sub-sections. Figure 2 illustrates the chemical structures of the different lipid classes with antimicrobial activity isolated from natural sources.

### 2.1. Extraction and Isolation of Plant Lipids

Studies that extract or isolate lipids from plants to test their antimicrobial activity are scarce (Table 1). The biomass used to extract lipids includes leaves, fruits, seeds, stems, rhizomes, shoots, stem barks, and heartwoods (Table 1). Lipid extraction from plants is usually carried out with organic solvents of different polarities (mainly *n*-hexane, CHCl_3_, CH_2_Cl_2_, EtOAc, EtOH, BuOH, MeOH, and their mixtures) (Table 1). Liquid/liquid extractions or Soxhlet extraction are commonly performed to obtain total lipid extracts [54,55,56,57,58]. Instead of analyzing one lipid class or one lipid molecule, some studies have analyzed the total lipid extracts that were obtained by sequential extraction.

In order to obtain a class of lipids or a particular lipid, the total lipid extract can be fractionated into different groups of lipids, depending on the polarity of the compounds, by thin-layer chromatography (TLC) or by column chromatography. Thus, for example, to recover the neutral lipids (e.g., sterol esters and triacylglycerols) by column chromatography, the extract can be eluted with CHCl_3_, followed by acetone to elute the glycolipids and, finally, with MeOH to elute the phospholipids, as mentioned for the leaves, stems and fruit of *Zygophyllum oxianum* [59]. The majority of the studies on antimicrobial plant lipids obtained and analyzed FA and their derivatives. FA have been isolated from a series of plant parts by using MeOH/benzene/sulfuric acid, 85% ethanol or supercritical fluid extraction (SFE) with CO_2_ and analyzed as FAME [56,60,61,62,63,64].

Mixtures of FA and FAME were obtained, but it was not clear if these FA were found in the free or esterified forms, since the derivatization methods (methylation) used in these studies convert free and esterified FA to FAME. However, because of their high abundance, presumably, the referred FA were esterified to other lipids. Several oxylipins were retrieved from roots, stems, and leaves of Brazilian joyweed (*Alternanthera brasiliana*) by extracting with EtOH and EtOAc [65]. Acetylenic FA were isolated from the roots of *Pentagonia gigantifolia* with 95% ethanol [38].

Other lipid classes isolated from plants for antimicrobial testing include sterols and sterol derivatives, glyceroglycolipids, and sphingolipids (Figure 2 and Table 1). The first group includes free sterols, steryl glycosides, and acyl steryl glycosides. Free sterols have been extracted together with triterpenes from the seeds of date palm (*Phoenix dactylifera*) by using CHCl_3_ and acetone [66], from several parts of *Withania somnifera*, *Euphorbia hirta*, and *Terminalia chebula* with EtOAc [67] and leaves of candle bush (*Senna alata*) and rhizomes of fingerroot (*Kaempferia pandurata*) with EtOH [68]. *β*-sitosterol 3-*O*-*β*-D-glucopyranoside, a steryl glycoside, was obtained from the leaves of Sendudok (*Melastoma malabathricum*) with CHCl_3_/acetone/MeOH [69] and the acyl steryl glycosides sitosteryl 3-*β*-*O*-glucoside 6’-*O*-palmitate and stigmasteryl 3-*β*-*O*-glucoside 6’-*O*-palmitate were obtained from the roots of capotiraguá (*Blutaparon portulacoides*) with EtOH [70]. Glyceroglycolipids, namely sulfoquinovosyldiacylglycerols (SQDG) were retrieved from neem (*Azadirachta indica*) leaves by extracting with MeOH and separating by SiO_2_ column chromatography with CHCl_3_/MeOH [71] or extracted with petroleum ether and re-extracted with MeOH [72].

In the group of sphingolipids, different chemical structures were identified belonging to different subclasses: ceramides and glycosphingolipids, also known as cerebrosides (Figure 2 and Table 1). Artemceramide-B was identified from the roots of *Artemisia incisa* after extraction with MeOH and recovered by SiO_2_ column chromatography after elution of the extract with CH_2_Cl_2_/MeOH following previous elution with *n*-hexane/EtOAc [73]. A cerebroside (soya-cerebroside I), a sphingolipid glycoside (1-*O*-*β*-D-glucopyranosyl(2*S,*3*S,*4*R,*10*E*)-2-[(2’*R*)-2-hydroxytetracosanoylamino]-1,3,4-octadecanetriol-10-ene), and its aglycone form (2*S,*3*S,*4*R,*10*E*)-2-[(2’*R*)-2-hydroxytetra-cosanoylamino]-1,3,4-octadecanetriol-10-ene) were isolated from the stems of cucumber (*Cucumis sativus*) by CHCl_3_ fractionation of the methanolic extract [74]. New glycosphingolipids were isolated and characterized from the fruits of fiddle leaf fig (*Ficus pandurata*), panduramides A–D and newbouldiamide [75], and from the woods of the giant-leaved fig (*Ficus lutea*), 1-*O*-*β*-D-glucopyranosyl-(2*S,*3*R,*5*E,*12*E*)-2*N*-[(2′*R*)-hydroxyhexadecanoyl]-octadecasphinga-5,12-dienine, commonly named lutaoside [76]. All these compounds are inhibitors of microbial growth, except panduramides A–D and newbouldiamide that did not reveal any activity (Table 2).

The fractionation of the extracts has been usually carried out by column chromatography and the purification of the compounds can be achieved by semi-preparative HPLC.

Different analytical platforms have been used to identify and characterize the structure of lipids in plant extracts. Generally, in natural products research, several complementary methods are used, such as ^1^H and ^13^C nuclear magnetic resonance (NMR) spectroscopy, gas chromatography (GC) coupled to a flame ionization detector (GC-FID) or to a mass spectrometer (GC-MS), as well as MS with electrospray ionization (ESI-MS). Liquid chromatography-MS (LC-MS) and LC-MS/MS has not been much used on antimicrobial plant lipids’ research, only for sphingolipids analysis [74] and for linoleate oxylipins’ structural characterization [65]. Besides these common techniques, two-dimensional NMR techniques (2-D NMR such as correlation spectroscopy-COSY, nuclear Overhauser effect spectroscopy-NOESY, heteronuclear single quantum coherence-HSQC, and heteronuclear multiple bond correlation-HMBC) have been used for the identification of artemceramide-D [73] and glyceroglycolipids [72]. Other methods are regularly used for the analysis of lipid extracts or their fractionation, such as TLC [57,69,74,77], column chromatography as mentioned above, or paper chromatography, but the information is very limited [69]. Analysis of FA is mostly performed by GC-FID or GC-MS, after derivatization. Total lipid extracts or lipid fractions are subjected to derivatization techniques using acid or alkaline hydrolysis or transmethylation to obtain FAME.

To identify and/or quantify mixtures of compounds, simpler techniques can be applied as biochemical assays using colorimetric tests, as for instance, for sterols’ and steryl glycosides’ identification and quantification [68,69,72]. The data obtained for compounds’ identification in these studies on antimicrobial plant lipids are normally compared with data reported in the literature, especially for spectroscopic data [72,83].

### 2.2. Susceptibility Testing, Inhibitory, and Microbicidal Activities of Plant Lipids

Several microbial strains were used in plant lipid studies, comprising Gram-positive bacteria, Gram-negative bacteria, acid-fast bacteria, yeasts, filamentous fungi, parasitic protozoa, and some MDR strains and/or hospital isolated strains, such as MRSA (Table 2).

The lowest MIC against *S. aureus* were observed for the mixture of lipid classes from the aerial parts of *Hedyotis pilulifera* (1.25 µg/mL) [83], the artemceramide-B from the roots of *A. incisa* (0.0313 mg/mL) [73], the linoleate oxylipins isolated from *Alternanthera brasiliana* (50 µg/mL) [65], and the FAME extracted from the shoots of *Salicornia brachiata* (60 µg/mL, the same MIC also verified for a MRSA strain) [61]. In the case of artemceramide-B, its high inhibitory potential against *S. aureus* was assigned to this polar lipid bearing four hydroxyl groups and an amide linkage between two long aliphatic chains [73]. In the case of the linoleate oxylipins from *A. brasiliana* plant tissues, five isolated oxylipins were also found to be synthesized by endophytic *Bacillus* strains isolated from this plant. So, it was speculated that the antimicrobial activity of the oxylipins from this plant could be derived from the endophytic bacteria, supposing an ecological crosstalk between this plant and its endogenous microbiome [65]. Also, the LC-MS/MS approach was crucial to identify these antimicrobial compounds both in the plants and in the bacteria, shedding some light on the plant–bacteria interplay [65].

The FA from the extract of *Cassia tora*’s leaves and stems exhibited MIC between 200 and 1000 μg/mL against MRSA [55]. The minimum bactericidal concentrations (MBC) against MRSA were determined on FAME from the leaves of *Excoecaria agallocha* (0.25 mg against *S. aureus*) [62], for MRSA the leaves of *Sesuvium portulacastrum* (1.0 mg/mL) [79], and the hexadecanoate methyl and hexadecanoic acid <*n*-> obtained from the stem bark of *Scaphium macropodum* (3.13 mg/mL against *S. aureus*) [82].

Other studies have tested the antimicrobial activity of lipids against other pathogenic microorganisms of great relevance for the clinical area and included in WHO’s guidelines, such as those of the “ESKAPE” group. The lipid extracts with greater inhibiting capacity over *Escherichia coli* were the FA and their derivatives from *n*-hexane and CHCl_3_ extracts of the heartwood of *A. adianthifolia* (1 µg) [79], and the acyl steryl glycosides obtained from roots of *B. portulacoides* (50 µg/mL) [70]. Also with low MIC values, the FAME extracted from the shoots of *S. brachiata* (0.5 mg/mL) [61] and the FAME and steroids from *Trigonella foenum-graecum* seeds (100 µg/mL) [63]. A MBC range between 1.0 and 2.0 mg/mL was verified for FAME extracts from the leaves of different plants [60,61,62].

Some lipid extracts were found to have low MIC against *P. aeruginosa*, as the FA and their derivatives obtained from the *n*-hexane and CHCl_3_ extracts of the heartwood of *A. adianthifolia* (50 µg) [79], FAME and steroids from fenugreek seeds (*T. foenum-graecum*, 100 µg/mL) [63], and the SQDG extracted from the neem leaves (*A. indica*, 128 µg/mL) [71]. MBC toward *P. aeruginosa* between 0.1 and 2.0 mg/mL were verified for the extracts of FAME from leaves of different plants [60,61,62], similarly to the findings for the *E. coli* strains.

For strains of *Salmonella typhimurium*, a high MIC value of 25 mg/mL was obtained from the stem bark extract of *S. macropodum* which contained four compounds including two FA (hexadecanoate methyl and hexadecanoic acid <*n*->) [82]. The SQDG extracted from the neem leaves showed antimicrobial activity against MDR strains of *Salmonella typhi* and *Shigella dysenteriae*, both with a MIC range between 32 and 64 µg/mL, and also against MDR strains of *E. coli* (64–128 µg/mL), *P. aeruginosa* (128 µg/mL), *S. aureus* (128–256 µg/mL), and *K. pneumoniae* (256 µg/mL) [71]. Also, identical MIC values (0.5 mg/mL) of FAME extracts [60,61,62] and FA [54] from different plants were observed against *K. pneumoniae*.

Studies with *Mycobacterium* sp. demonstrated antimicrobial activity of extracts of fenugreek seeds that contained a mixture of FAME and steroids, having a MIC of 100 µg/mL against *M. tuberculosis* [63]. Activity against *Mycobacterium smegmatis* was also verified by extracts containing hexadecanoate methyl and hexadecanoic acid <*n*-> FA from the stem bark of Malva nut, *S. macropodum* (3.13 mg/mL) and a MBC of 6.25 mg/mL [82]. Finally, the triterpenoids oleanolic acid and rotungenic acid were found to have activity against *M. smegmatis* with a MIC of 2.5 µg/mL and 1.25 µg/mL, respectively [83].

The fenugreek seed extracts from which conjugated linoleic acid methyl ester, saturated FAME, and steroids were identified, showed an inhibitory effect against *Plasmodium falciparum* with a MIC of 0.29 µg/mL [63].

The glycolipid SQDG isolated from neem has a broad-spectrum of activity against MDR bacterial strains [71] and anti-helminthic activity [72], which proves to be a promising natural antimicrobial agent. This class of compounds isolated from neem has demonstrated antiviral activity (herpes simplex viruses, HSV-1 and HSV-2) [71], significant DNA binding properties [84], and anti-leukemic activity [85]. However, it is difficult to isolate a single compound or a class of compounds from complex matrices as plants. In most studies, the antimicrobial effects may be due to a synergistic effect between several natural antimicrobial compounds, and not just to the referred lipids. As such, more studies must be done to understand which lipids can effectively be responsible by the inhibitory or microbicidal effect and the structure–activity relationship.

## 3. Antimicrobial Lipids from Marine Organisms

The most studied antimicrobial compounds of marine origin are peptides and alkaloids [86,87,88], contrarily to lipids. However, lipids are ubiquitously distributed in the different marine phyla, being quite abundant in some of them. Besides, several lipid classes from marine organisms have been recognized by their biological activity with a high potential to discover new antimicrobial compounds.

### 3.1. Marine Algae

Algal biomass is mainly composed by minerals, sugars, proteins, and lipids, that represent 1–15%, depending on the algal species and its habitat. Lipids found in the macroalgae from the three phyla, Rhodophyta, Chlorophyta, and Ochrophyta (Table 3), have demonstrated antimicrobial activity against Gram-positive and Gram-negative bacteria, yeasts, and fungi [29,89,90,91,92] (Table 4). Most of these antimicrobial lipids were isolated from Rhodophyta and Chlorophyta. While the former shows a high diversity of algal species as source of antimicrobial lipids, studies in Chlorophyta were focused on species belonging to the order Bryopsidales.

Several studies have tested the antimicrobial activity of macroalgal extracts obtained with different solvents. Shanab (2007) compared extracts from three macroalgae species (*Sargassum dentifolium*, *Laurencia papillosa,* and *Jania corniculata*) using two solvents, EtOH and CH_2_Cl_2_ [93]. Both extracts exhibited similar antimicrobial activity against all microorganisms tested (bacteria and yeasts), except against the mold *Aspergillus flavus* [93]. Several extracts from *Gracilaria gracilis* were studied to identify potential bioactive compounds [94]. CHCl_3_ and Et_2_O extracts (apolar solvents) presented lower extraction yields (% of dry algal biomass) than extracts from more polar solvents (EtOH, MeOH, and acetone). Less polar solvents isolated minor lipid classes (e.g., neutral and medium polar lipids) and showed lower amounts of soluble carbohydrates and total phenols than polar solvents. However, the diameter of the inhibition zones against *B. subtilis* were slightly lower in these extracts than in the extracts obtained with polar solvents (rich in soluble carbohydrates and phenolic compounds) [94]. These results suggest that although less polar solvents have lower yields, they contain compounds with interesting antibacterial features.

The lipids or lipid mixtures, their extracting solvent(s), and the methods used for their characterization in algae species are summarized in Table 3. The results of the antimicrobial assays with lipids and lipid-rich extracts from these algae are summarized in Table 4. Chemical structures of lipids isolated from algae are represented in Figure 2.

#### 3.1.1. Fatty Acids

FA have been commonly reported in algae as antimicrobial agents. However, most studies tested total extracts instead of lipid-rich fractions and the antimicrobial activity was usually assigned to the most abundant FA in the extract [96,105,116]. The lack of target analyses limits the interpretation of the findings because FA are usually low abundant in total lipid extracts in the free form, being mainly esterified to other lipids, such as polar lipids, sterols, and triacylglycerols.

The antibacterial activity of the extracts from the microalga *Dunaliella salina* was suggested to be dependent on the presence of palmitic (16:0), 9,12,15-octadecatrienoic (α-linolenic, 18:3 *n*-3) and 18:1 *n*-9 together with volatile compounds [123]. The antimicrobial activity of the red macroalgae, *J. corniculata,* and *L. papillosa*, was ascribed to the presence of saturated [14:0, 16:0, stearic (18:0) acids] and the UFA [palmitoleic (16:1), 18:1 and nervonic (24:1) acids] [93]. Also, the antimicrobial activity of *S. dentifolium* was associated with the high relative abundance of 18:0, nonadecanoic (19:0), arachidic (20:0), petroselinic (18:1 *n*-12), and 24:1 acids [93]. Plaza et al. (2010) associated the antimicrobial and antifungal activities of the alga *Himanthalia elongata* and the microalga *Synechocystis* sp. with the high amount of 16:1 *n*-7 and 18:1 acids in the extracts, respectively [116]. The FA 18:3 was identified as an antibacterial agent in *Chaetomorpha linum* against *Vibrio ordalii* and *V. vulnificus* [99]. A relationship was found among the antimicrobial activity against *S. aureus*, *E. coli,* and *C. albicans*, and the FA docosapentaenoic (DPA, 22:5) and triacylglycerol contents [121]. In the green macroalga *Ulva rigida*, purified fractions of crude extracts with the highest antibacterial activity against *S. aureus* and *Enterococcus faecalis* comprised mainly 16:0, 18:1, and 16:1 *n*-7 FA [27]. Nevertheless, most of these studies neither isolated the predominant FA nor tested its activity. The FA 16:0 was the most abundant FA in *Gracilariopsis longissima*’s lipid extracts, that presented antibacterial activity against several *Vibrio* species. However, the pure FA 16:0 did not show any antibacterial activity [108]. On the other hand, some studies have isolated, purified, and characterized the FA with potential antimicrobial activity. For instance, marine diatoms revealed to be a rich source of FA with antibacterial properties. Studies with the diatom *Navicula delognei* allowed the isolation and identification of two FA (6*Z*,9*Z*,12*Z*,15*Z*)-hexadecatetraenoic acid (16:4 *n*-1) and (6*Z*,9*Z*,12*Z*,15*Z*)-octadecatetraenoic acid (18:4 *n*-3) and an ester (*E*)-phytol(5*Z*,8*Z*,11*Z*,14*Z*,17*Z*)-eicosapentaenoate with potent antibacterial effect against *S. typhimurium*, *S. aureus,* and *S. epidermidis* [124]. Two FA with antibacterial features were isolated from the marine diatom *Phaeodactylum tricornutum*, (9*Z*)-hexadecenoic acid (16:1 *n*-7), and (6*Z*,9*Z*,12*Z*)-hexadecatrienoic acid (HTA, 16:3 *n*-4) [125]. Both FA inhibited the growth of *S. aureus* and *S. epidermidis* [125]. Furthermore, 16:1 *n*-7 showed a potent antibacterial activity against two MRSA strains, and HTA against the Gram-negative marine bacterium *Listonella anguillarum* [125]. From the same diatom species, the eicosapentaenoic acid (EPA, 20:5 *n*-3) was isolated and tested, inhibiting the growth of Gram-negative and Gram-positive bacterial species, including MRSA [126]. The presence of double bonds in C16 FA was suggested to be crucial for their antibacterial action [125]. A higher (two-fold) inhibitory effect against *S. aureus* was recorded for 16:1 *n*-7 in relation to HTA isolated from the marine diatom *P. tricornutum* [125]. Nevertheless, in the macroalga *Sargassum pallidum*, free FA fractions having higher proportion of UFA demonstrated to be more active against more microorganisms like *S. aureus*, *C. albicans*, *A. niger,* and *Septoria glycines* [118]. Five UFA ethyl esters were isolated from the red macroalga *Laurencia okamurai*: (9*Z*,12*Z*,15*Z*,18*Z*,21*Z*)-ethyl tetracosa-9,12,15,18,21-pentaenoate, (10*Z*,13*Z*)-ethyl nonadeca-10,13-dienoate, (9*Z*,12*Z*)-ethyl nonadeca-9,12-dienoate, (*Z*)-ethyl octadec-13-enoate, and (*Z*)-ethyl hexadec-11-enoate [109]. All FA ethyl esters revealed individual antifungal activity against *Candida glabrata*, four of them against *Cryptococcus neoformans* and two against *Trichophyton rubrum* [109].

Although several studies have suggested that the FA chain length and double bond position influence their antimicrobial activity, there is no unanimity on the correlation between PUFA and antimicrobial activity [127].

#### 3.1.2. Glycolipids

Glycolipids are the predominant lipids in the chloroplasts’ membranes of plants, eukaryotic algae, and cyanobacteria. They are recognized by their essential role in photosynthesis and by their structural functions as major components of the thylakoid membranes [128,129]. Algae synthesize two major types of glycolipids: neutral galactolipids and negatively charged sulfolipids. Neutral galactolipids include monogalactosyldiacylglycerol (MGDG), digalactosyldiacylglycerol (DGDG), and their lyso forms [monogalactosylmonoacylglycerol (MGMG), and digalactosylmonoacylglycerol (DGMG)]. Sulfolipids include SQDG and its lyso form sulfoquinovosylmonoacylglycerol (SQMG).

Glycolipids have been identified as biogenic compounds possessing a variety of bioactivities, such as antioxidant, antiviral, or antitumor [89,95,103,122,130,131,132]. The biological activity of glycolipids has been associated to the length of their fatty acyl chains, the number and position of the double bonds, the structure of the sugar moiety, and its anomeric configuration [131]. Gerasimenko et al. (2014) characterized the FA profiles and the antimicrobial activity of glycolipid fractions from *S. pallidum* throughout the year but could not assign any relationship between the amount of UFA and antimicrobial activity of DGDG, MGDG, and SGDG fractions [118]. However, a dependent effect of SFA level was observed in the antimicrobial activity of SQDG and MGDG fractions [118].

Several studies identified glycolipid fractions from algae having antimicrobial activity [89,103], even though only a few could isolate and characterize the main molecular species responsible for this activity. SQDG(20:5/16:0) was isolated from the red macroalga *Gigartina tenella*, which inhibited HIV-1 reverse transcriptase [107]. The sulfolipid 1-*O*-palmitoyl-3-*O*(6′-sulfo-α-quinovopyranosyl) glycerol (SQMG 16:0) was isolated from the methanolic extract of the brown seaweed *Sargassum wightii* and its activity was tested against *Xanthomonas oryzae* pv. *oryzae* [120]. The sulfolipid (2*S*)-1,2-di-*O*-palmitoyl-3-*O*-(6′-sulfo-α-D-quinovopyranosyl) glycerol, SDGQ(16:0/16:0), from the green alga *Caulerpa racemosa* was found to be a potent anti-HSV-2 agent [95]. An enriched sulfolipid fraction obtained from the red macroalga *Osmundaria obtusiloba* demonstrated potent antiviral activity against the herpes viruses HSV-1 and HSV-2, being the SQDG(14:0/16:0) the most abundant molecular species identified in that fraction [111]. However, that sulfolipid-rich fraction had a lower antiviral activity than the crude MeOH fraction, maybe due to synergistic effects with other glycolipid species, such as MGDG and DGDG [97,111]. SQDG (14:0/16:0) was identified in the glycolipid-rich extracts from the brown seaweed *Sargassum vulgare* as the main responsible for their anti-HSV-1 and anti-HSV-2 activity [119]. Promising results were recorded in the antibacterial activity of the glycolipid-rich fraction from the brown macroalga *Fucus evanescens* against *P. acnes,* MGDG (18:3/18:3) being the main active compound [115]. The authors synthesized this glycolipid and replicated the bioassay, but its inhibition efficiency toward *P. acnes* was lower (50%) than the glycolipid-rich fraction (>99%) [115], suggesting a synergistic antibacterial effect with other glycolipid compounds. Additionally, the antiviral activity of SQDG and SQMG isolated from *Ulva fasciata, Laurencia papillosa, Galaxoura cylindrica, Dictyota fasciola,* and *Taonia atomaria* was shown against HSV-1. This activity was attributed to the high concentration in these glycolipids [89]. The sulfolipid fraction also demonstrated a high inhibition against *E. coli* and *B. subtilis*, being the extracts from *U. fasciata* and *T. atomaria* those with the highest inhibitory capacity [89]. The potent antiviral effect of sulfolipids has been related with the negative charge of their sulfonate group as verified for marine polysaccharides [133].

Glycolipids from the red alga *Chondria armata* were recognized by their antibacterial and antifungal activities against several pathogens, such as *C. albicans* and *C. neoformans* or *Klebsiella* sp. [104]. The main bioactive lipid from the glycolipid fraction was identified as 1-eicosapentanoyl-2-palmitoyl-3-*O*-galactopyranosyl-glycerol, MGDG(20:5/16:0) [104]. The isolated fractions of MGDG and SQDG obtained from the lipophilic fractions of the brown seaweed *Laminaria cichorioides* inhibited the growth of the yeast *Safale* and *C. albicans*, the fungi *A. niger* and *F. oxysporum*, and the bacteria *S. aureus* and *E. coli* [91].

Several studies demonstrated higher antimicrobial activity in lipid-rich fractions than in total lipid extracts [91,118]. In the brown seaweed *S. pallidum*, the isolated fractions of glycolipids and free FA demonstrated to be more efficient in inhibiting bacterial growth than the total lipid extract [118]. The antimicrobial activity efficiency may be conditioned by seasonal variation, that must be related with shifts in the lipid profiles promoted by alterations in growth-related environmental conditions [118]. Nevertheless, a seasonal variation was not always verified, like in the green seaweed *U. rigida* that demonstrated a uniform antimicrobial activity throughout the year [27].

#### 3.1.3. Other Lipids

The exploration of algal lipidomes has revealed a panoply of compounds with antimicrobial activity. Two compounds with antiviral properties against Semeliki forest and Japanese encephalitis viruses were isolated from *U. fasciata*’s lipophilic fractions, a sphingosine (*N*-palmitoyl-2-amino-1,3,4,5-tetrahydroxyoctadecane) and a ceramide (erythro-sphinga-4,8-dienine-*N*-palmitate), respectively [101,102]. Two bromoditerpenes, with antibacterial and antimalarial activity against *S. aureus* and the chloroquine-resistant *P. falciparum*, respectively, were isolated from the red alga *Sphaerococcus coronopifolius* [114]. Four halogenated sesquiterpenes isolated from the surface of the red macroalga *Laurencia* spp. demonstrated antibacterial activities toward six Gram-negative bacteria such as *V. parahaemolyticus*, *Chromobacterium violaceum,* or *Erwinia sp*. Neophytadiene and phytol were identified in the microalga *D. salina* as putative antimicrobial compounds against *E. coli*, *S. aureus*, *C. albicans*, and *A. niger* [123]. Several terpenes isolated from the brown seaweeds *Sargassum fusiforme* and *S. vulga*re were identified as putative antimicrobial molecules against clinical bacteria [117]. Terpenes were also suggested as antibacterial compounds against MRSA and *E. coli* in the red seaweeds *Caulerpa racemosa* and *Caulerpa lentillifera* [96].

Clerosterol was the main compound identified in a bioactive fraction from the green seaweed *Codium amplivesiculatum* against Gram-positive (*S. aureus*) and Gram-negative (*V. parahaemolyticus*) bacteria [100]. However, this compound did not display antibacterial activity when tested alone [100]. Different phospholipid classes, such as phosphatidylethanolamine and phosphatidylserine were identified in the red seaweed *Pyropia orbicularis*, as putative antimicrobial compounds, together with MGDG [113].

#### 3.1.4. Photosynthetic Pigments

Pigments are not recognized as lipids, but they are present in the total lipid extracts of algae since they are extracted from the biomass along with lipids. Besides, in most studies, they were not removed from the lipid extracts. Pigments are mainly recognized by their antioxidant activity [134,135], but some studies have attributed a weak antibacterial activity to photosynthetic pigments such as chlorophylls and carotenoids [93]. Pheophytin α and chlorophyllide α from the microalga *Isochrysis galbana* were found to have a high antibacterial activity against marine strains of *Brevibacterium* and *Micrococcus* [136]. Carotenoid derivatives (β-cyclocitral and α- and β-ionone) from the microalga *D. salina* extracts were suggested to be responsible for its antimicrobial activity [123]. Fucoxanthin-rich fractions from the brown macroalga *L. cichorioides* demonstrated weak activity against yeast (*Safale* and *C. albicans*) and bacteria (*S. aureus* and *E. coli*), while chlorophyll fractions showed antibacterial activity against *S. aureus* and *E. coli* [91]. Photosynthetic pigments isolated from the brown macroalga *S. pallidum*’s extracts presented antifungal and antibacterial activities, chlorophylls (chlorophyll α as the main component) being the mediator to inhibit the growth of the fungi *S. glycines* and *A. niger*, while fucoxanthin was effective against *E. coli* [118].

### 3.2. Marine Invertebrates

The chemotaxonomic diversity of marine invertebrates is responsible for the large number of novel compounds identified in their phyla. Tropical biodiversity-rich benthic communities have been the most explored, thus the most fruitful in the identification of new potential antimicrobial compounds [137,138,139]. However, less conventional environments such as the Arctic ocean or mesopelagic communities have been started to be surveyed [140,141].

Marine invertebrates comprise a growing source of natural compounds, showing novel structures for biomedical and health-promoting applications [142]. Bioprospection of new compounds from marine invertebrates has revealed to be a prolific work to discover diverse bioactive compounds with action toward a broad spectrum of microorganisms [140,143]. Some of these reports have identified total lipid extracts as a potential source of bioactive compounds, lacking a sequential workflow of isolation, characterization, and purification of the metabolites responsible for the activity [99,143]. Although most of these studies used classical bioprospection methods to identify the bioactive compounds from marine species, others followed eco-friendly approaches by using fishing waste [141] or seafood by-products [144].

Phyla of marine invertebrates recognized as sources of antimicrobial compounds [16,145] include porifera [146,147], crustacean [148,149], mollusk [144,150,151], or cnidaria [137,139]. Some bacteria isolated from marine organisms have also disclosed antibacterial activity, such as *Actinobacteria* from sponges [26].

The main antibacterial natural products identified in marine invertebrates were peptides, polyketides, alkaloids, terpenes, and lipopeptides [14,34,150,152]. However, several antimicrobial lipids classes have been identified. Marine invertebrates produce an array of unique lipids originating from unusual biosynthetic pathways that are not common in other environments, as a result of thriving in diverse and extreme environments [142,153].

Porifera represents the most studied phylum of marine invertebrates for antimicrobial compounds’ bioprospection, including lipids [154,155]. The high contribution of these ancestral metazoans for bioactive compounds’ research seems to be related to their high filtering activity, pumping water during feeding, which expose them to viruses, bacteria, and eukaryotic organisms (pathogenic and non-pathogenic) [156,157].

Table 5 assembles the information regarding lipids from marine invertebrate species having antimicrobial activity. Table 6 summarizes the information about the antimicrobial properties, the tested organisms, and the antimicrobial assays for each marine invertebrates’ species listed in Table 5. Figure 2 illustrates the chemical structure of the main lipid classes with antimicrobial properties from these natural sources.

#### 3.2.1. Fatty Acids

FA identified in marine invertebrates display a high diversity of chemical structures that are scarce or inexistent in other environments, such as very long chain PUFA or cyclic forms [158,159,160]. A C14 acetylenic FA isolated from the sponge *Oceanapia* sp. revealed antimicrobial activity against nine microbial strains, including yeasts and the bacteria *E. coli*, *P. aeruginosa*, *B. subtilis*, and *S. aureus* [161]. Complex FA mixtures containing very long chain FA were isolated from the sponge *Agelas oroides* and tested as anti-infectious agents [162]. These FA inhibited the enoyl reductases, enzymes that catalyze the last step of the elongation cycle in FA synthesis, in *P. falciparum*, *M. tuberculosis,*, and *E. coli* [162]. An acetylenic FA having antibacterial activity against *S. aureus* and *E. coli* was discovered in the marine sponge *Paragrantia* cf. *waguensis* [163]. Ravichandran et al. (2010) suggested 18:1 and 18:2 FA as antimicrobial compounds from the hemolymph and hemocytes of brachyuran crabs with greatest activity against *V. cholerae*, *S. flexneri*, *S. pyogenes*, and *E. coli* [148]. Brominated FA from a sponge of the genus *Xestospongia* showed antimicrobial activity against MRSA, *S. mutans*, and *S. sobrinus* [138]. Anti-infective brominated long-chain acids, termed motualevic acids, and an enantiomer of antazirine, were isolated from the sponge *Siliquariaspongia* sp. with activity against *S. aureus* and MRSA [164]. A recent study screening mesopelagic species as a source of potential antimicrobial compounds recognized the extracts of the lanternfish *Myctophum punctatum* and the Mediterranean krill *Meganyctiphanes norvegica* to inhibit the growth of MRSA, MSSA, and *M. tuberculosis* [141]. Extracts of both marine species were fractionated, and their composition was elucidated by LC-UV analysis. EPA, 4,7,10,13,16,19-docosahexaenoate (DHA, 22:6 *n-*3) and 8,11,14,17-eicosatetraenoic (ETA, 20:4 *n*-3) acids were the most abundant components in the lipid fractions of both marine species [141]. The 14-methyl-5,9-pentadecadienoic FA from the phospholipid pool was isolated and identified in the gorgonian coral *Eunicea succinea* and it was active against *S. aureus* and *E. faecalis* [137]. Quantitative data on antimicrobial efficiency are shown in Table 6.

#### 3.2.2. Sterols

Sterols with singular structures were isolated from marine invertebrates, exhibiting novel carbon skeletons. Marine sponges were found to be sources of steroid sulfates with antiviral activity against HIV [165,166] and against feline leukemia virus (FeLV) [166] and antifungal activity against the yeasts *C. albicans* [167,168] and *S. cerevisiae* [168,169]. Three sterols were isolated and identified in the marine sponge *Haliclona simulans* with anti-mycobacterial and anti-trypanosomal activity [170]. The EtOAc extract of the flesh of the rock oyster *Saccostrea glomerate* was fractionated, and the best active fraction showed several sterols (e.g., cholesterol, stigmasterol, sitosterol) and FA as putative active compounds with antibacterial and antifungal activities against a broad spectrum of microorganisms [171]. Steroidal glycosides (e.g., eryloside, wondosterols, sarasinoside, sokodosides) isolated from marine sponges also showed antimicrobial properties [172,173,174,175].

#### 3.2.3. Polar Lipids

Polar lipids are the main constituents of biological membranes and are also present in biofluids. The methanolic extracts of both the hemolymph (plasma) and hemocytes (plasma cells) of six brachyuran crabs revealed antimicrobial activity against sixteen bacterial and fungal pathogenic strains [148]. The antimicrobial activity of the hemolymph extracts was assigned to the presence of polar lipids [148].

A new family of antimicrobial glycolipids, caminosides, isolated from the marine sponge *Caminus sphaeroconia* showed a potent in vitro inhibition against a panel of human and plant pathogens (e.g., MRSA, vancomycin-resistant *Enterococcus*) and enteropathogenic *E. coli* (EPEC) [147,176]. Several ceramide and glycolipid molecular species have been isolated from soft corals of the genus *Sinularia*, and exhibited antibacterial and antifungal activity against seven microbial strains [139]. A ceramide from the cnidaria *Lobophytum crassum* showed moderate antibacterial activity against *P. aeruginosa*, *S. epidermis*, *B. subtilis,* and *S. aureus* [177].

Six lysophospholipids (lyso-platelet-activating factor, PAF) isolated from the sponge *Spheciospongia purpurea* displayed moderate antifungal activity against *C. neoformans*, *C. glabrata*, *T. rubrum*, and *A. fumigatus* [146]. The lyso-PAF molecular species were identified as PAF(16:0), PAF (16:1 *n*-5), PAF (18:0), PAF (18:1 *n*-7), PAF (18:1 *n*-11), and PAF (18:1 *n*-13) [146]. Two lyso-PAF, 1-*O*-hexadecyl-*sn*-glycero-3-phosphocholine and 1-*O*-octa-decyl-*sn*-glycero-3-phosphocholine, from the demosponge *Suberites domuncula* were separated and purified by reverse-phase (RP) HPLC, with further characterization by FIA-MS, LC-MS, and ESI-MS [157]. These compounds demonstrated a potent antibacterial activity against the bacterium SB1, isolated from *S. domuncula*, which presented a high species-level similarity (>98%) to the α-*Proteobacterium* MBIC3368 [157].

#### 3.2.4. Other Lipids

Diverse structural modifications of isoprene units provide terpenes with a large range of biological activities [34]. These compounds were isolated mainly from Demospongiae species [178,179,180,181,182] and displayed antimicrobial activity against several infectious agents [183,184,185,186]. Six sesquiterpenoids, termed halichonadins, isolated from the marine sponge *Halichondria* sp. showed antimicrobial activity against the bacterium *M. luteus*, the yeast *C. neoformans,* and the mold *Trichophyton mentagrophytes* [187,188]. A cembranoid diterpene isolated from the cnidaria *L. crassum* showed strong antibacterial activity against *P. aeruginosa*, *S. epidermis*, *B. subtilis,* and *S. aureus* [177]. Two meroterpenoids, rossinones, from the Antarctic ascidian *Aplidium* sp. exhibited antiviral, antibacterial, and antifungal activities [189].

Terpenes with functional activities were identified in the cnidaria phylum, as from the soft coral *Antillogorgia elisabethae* [192]. Seven diterpenes showed potent antibacterial activity against the Gram-positive bacteria *S. pyogenes*, *S. aureus,* and *E. faecalis* [192]. A new series of lipids, termed mololipids, were found in the lipidome of a sponge from the order Verongida (recognized as “the Moloka’i sponge”) and were reported to be active against HIV-1 [191]. These lipids present a core moloka’iamine nucleus with two FA-derived side chains, saturated linear and iso-methyl branched ranging from 14 to 20 carbons [191].

#### 3.2.5. Pigments

Xanthophyll, *β*-cryptoxanthin, and *β*-carotene obtained from the flesh and coelomic fluid of *Holothuria scabra* (sea cucumbers) showed antibacterial activity against *S. aureus* [193]. Skin pigments of the jumbo squid *Dosidicus gigas* exhibited growth inhibition against several bacteria, fungi, and yeast species [144]. The highest inhibition zone diameter was verified against *S. enterica*, and ommatins, specifically of the xanthommatin type, were suggested as the main active compounds [144].

## 4. Cytotoxicity of Natural Antimicrobial Lipids against Mammalian Cells

Some lipid and lipid-rich extracts from natural sources showed high MIC and MBC values, in the range of mg/mL, as can be seen in Table 2, Table 4, and Table 6. This is more than one thousand times larger than is of pharmaceutical significance and raises an important question regarding cytotoxicity to mammalian cells and safety to human beings. Some studies emphasized the low toxicity to eukaryotic cells of antimicrobial lipids from natural sources, namely of FA [158]. But most studies that searched for natural lipids with antimicrobial activity did not perform in vitro or in vivo cytotoxicity experiments. Data regarding mammalian cytotoxicity are not yet available for most of these compounds, which is a limitation of the studies presented in this review about antimicrobial plant lipids. Most studies that tested antimicrobial and cytotoxicity activities from marine lipids are limited to lipid-rich extracts [145,194] or lipid-rich fractions [97,195]. For instance, petroleum ether fractions of *Grateloupia livida* extracts demonstrated a higher antibacterial activity and this fraction did not display acute oral toxicity in mice (100% survival rate at doses up to 2.0 g/Kg) [195]. Even so, careful conclusions should be taken from these evidences, since petroleum ether is a carcinogenic solvent. The MeOH and acetone fractions from *O. obtusiloba* demonstrated activity against HSV-1 and low toxicity to Vero cell (African Green monkey kidney cells) cultures, showing 50% cytotoxic concentration (CC_50_) of 172 and >200 µg/mL in MeOH and acetone fractions, respectively [97]. A sulfolipid-rich fraction from the same red macroalga showed a higher cytotoxicity with CC_50_ of 72 µg/mL in Vero cell lines [111]. SQDG fractions from *S. vulgare* with antiviral activity (HSV-1 and HSV-2), displayed CC_50_ > 200 µg/mL in Vero cells [119]. The ethanolic extract as well as the glycolipid, FA and pigments’ fractions from *L. cichorioides* demonstrated hemolytic activity at 200 µg/mL in erythrocytes of mongrel white mice [91]. SQDG(16:0/16:0) isolated from *C. racemosa* with antiviral activity (HSV-2) had very low toxicity to Vero cells ATCC CCL-81 with a CC_50_ of 1.0 g/mL [95]. An acetylenic FA isolated from the calcareous sponge *P*. cf. *waguensis* demonstrated weak cytotoxic effect against NBT-T2 rat bladder epithelial cells (IC_50_ > 20 mg/mL) [163]. FA mixtures isolated from the sponge *A. oroides* exhibited in vitro antiplasmodial, trypanocidal, and leishmanicidal activities with weak cytotoxicity toward mammalian (rat skeletal myoblast L6) cells (IC_50_ from 43 to >90 µg/mL) [162]. Sterols isolated from the sponge *H. simulans* demonstrated anti-mycobacterial and anti-trypanosomal activities, but low cytotoxicity on normal fibroblasts derived from human foreskin (Hs27) cells with IC_50_ ranges from 58 to >100 µM [170]. Meroterpene sulfate molecules isolated from the sponge *Fasciospongia* sp. was inactive (IC_50_ > 30 µM) toward human foreskin fibroblast (HFF-1) cell lines [186]. Finally, lipid extracts from different body compartments (hemolymph, hemocyte, eggs, muscle, and exoskeleton) of four marine crustaceans demonstrated hemolytic activity against human red blood cells, hemolymph and exoskeleton extracts being those that presented the highest activity [149].

Molecules, in this case lipids, with such high MIC values have little chance of undergoing clinical development. Nevertheless, the results of the reviewed reports should allow to focus on the most interesting molecules, having a low MIC value, and reserve the molecules with a high MIC value for other applications, such as veterinary clinic or agriculture.

## 5. Lipidomics for the Analysis of Bioactive Lipids in Plants and in Marine Organisms

The identification and characterization of lipids as natural antimicrobial agents is of utmost importance, either for their exploitation from natural sources or to understand their mechanisms of action. Lipid identification from natural sources is achieved by using lipidomic strategies. Lipidomics consists in the analysis, identification, and structural characterization of the lipid molecular species from a biological matrix, based on chromatography and MS techniques. It also involves optimizing extraction and fractionation processes, considering the polarity of the different lipids that make up the biological systems. As such, lipidomics is a valuable tool to discover bioactive lipids with antimicrobial activity.

**Figure 3:**
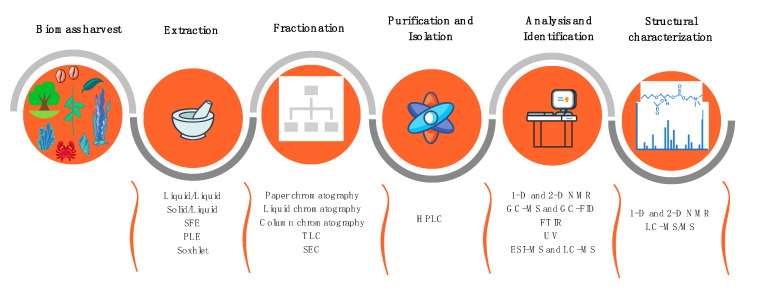
Workflow summarizing the main techniques used for lipidomics analyses of lipids from plants and marine organisms.

The main phases of the lipidomics workflow include lipid extraction, fractionation, and/or enrichment steps, and analysis of total extracts or lipid fractions by MS combined with LC and/or GC (Figure 3).

Lipid extraction is usually performed with organic solvents. Mixtures of CHCl_3_, MeOH, and H_2_O are the most used, such as the Bligh and Dyer [196] or Folch [197] methods and their modifications. These methods are considered the most efficient to extract the total lipid content. However, there are other solvents used for lipid extraction, such as EtOH, MeOH, *n*-hexane, BuOH, EtOAc, giving rise to different lipid yields [198,199]. CHCl_3_ is being replaced by CH_2_Cl_2_ in these methods because the latter is less toxic and allows a similar extraction efficiency. The solvent or the mixture of solvents used in the extraction step(s) exert a great influence in the lipid composition of the extracts obtained from natural sources. In most cases, extractions with different solvents lead to crude extracts with the same components but in different amounts. It is also possible to selectively extract some lipid classes, such as the extraction performed with apolar solvents, as *n*-hexane, which recover mostly apolar lipids (e.g., triacylglycerols). Nevertheless, an effective extraction is very important for the identification of bioactive lipids. Non-conventional methods of extraction as ultrasound- and microwave-assisted extraction or SFE [63] are used to help the extraction, particularly to improve the lipids yield, or to obtain extracts with specific functions and applications. Assisted extraction methods are frequently combined with green solvents, such as EtOH. Soxhlet-assisted extraction with less polar solvents, such as *n*-hexane, is normally used to isolate lipophilic and more apolar lipids, as terpenoids and sterols. Testing potential antimicrobial lipid extracts obtained with different solvents, even from the same source, would provide dissimilar bioactive properties due to different lipid compositions.

Total lipid extracts include a great diversity of lipid classes and molecular species. Fractionation of the total lipid extracts to obtain fractions enriched with specific lipid classes can be achieved by solid-phase extraction (SPE), using columns of simple silica (SPE-SiO_2_) or functionalized silica, by preparative HPLC, or by TLC, although the latter is less common nowadays. TLC can also be used as an identification tool in lipid extracts or fractions, by applying spots of well-known lipid standards in the TLC plate and compare their retention factor with the samples’ bands after elution. However, it has the disadvantages of lacking information at lipid molecular level and being time-consuming [200]. A careful choice of the eluents and gradients allows obtaining a selective separation of lipid fractions from the initial extract. The fractions obtained can be used for diverse purposes: to quantify the different lipid classes (e.g., by gravimetry), to separate neutral lipids (e.g., triacylglycerols and sterols) from polar lipids (e.g., phospholipids, glycolipids), or to identify different phospholipid classes, that can be further separated by TLC and estimated by phosphorus quantification of each TLC spot. The lipid fractions can be then used to evaluate the bioactive properties of a specific lipid class and analyzed by GC-MS and/or LC-MS to provide an insight on the structure–activity relationship.

The characterization of total lipids or lipid fractions is done, nowadays, by MS-based approaches using several kinds of mass spectrometers coupled with chromatography. GC-MS is used to identify and quantify FA after derivatization (usually methylation) of apolar lipids as terpenoids and sterols (after derivatization by silylation). GC-MS allows the identification of these molecules by specific retention times, mass spectra analysis, and data base comparison. Nevertheless, GC-MS neither covers large molecules nor non-volatile molecules. Thus, when this approach is used to characterize bioactive lipids, a lot of information is missing. LC-MS allows a broader coverage than GC-MS of lipid species and larger molecules, usually the most abundant ones in the lipid extracts, such as triacylglycerols, polar lipids, or esterified sterols. Besides, small molecules like free FA and free sterols are also detected.

LC-MS identifies the lipid molecular species by their retention time, accurate molecular weight observed in the mass spectra (i.e., LC-MS level), and detailed structural features by interpretation of their LC-MS/MS spectra. The chromatographic peaks are integrated to plot the quantification of each molecular species, which is normalized by an appropriate internal standard to reach a relative quantification. Routine lipidomics analyses use reversed-phase (e.g., C18 or C30), normal-phase, or hydrophilic interaction liquid chromatography (HILIC) columns [200]. In the former, the elution of the lipid molecules is based on their FA composition. So, an overlap of molecular species from different lipid classes but presenting the same FA composition can occur. In the latter two types of columns, the lipid molecules are separated by their hydrophilic properties, allowing to separate the different lipid classes present in the sample, which will depend on the polar head features.

Recent developments in MS technologies permit to acquire a huge amount of data in a short time frame, covering more than three hundred lipid species in one single LC-MS run. This can be done by means of the high-resolution mass spectrometers Orbitrap or quadrupole-time-of-flight (Q-TOF), available for untargeted or targeted analysis, or the triple quadrupoles (QQQ) and Q-traps, that are more suitable for targeted analysis, i.e., quantification of previously selected molecular species. However, data analysis is still time-consuming because of the lack of universal and exhaustive lipidomics databases and software. Some databases and software currently used are the LIPID MAPS database, the MZmine, LipidBlast, LipoStar, LipidSearch, and LPPtiger software.

The identification of bioactive molecules from natural sources is a laborious work that requires performing extraction, bioassays, fractionation, and in some cases, identification, in a sequential way. This is to achieve a specific class of bioactive metabolites or, preferentially, a unique and well-defined bioactive molecule. This process is called bioassay-guided fractionation and is emerging in the field of drug discovery from natural sources [201]. This approach usually starts with different extracts obtained by using solvents with different polarities, in order to have a set of extracts enriched in different types of metabolites. These extracts are evaluated for their putative bioactivities and the one(s) with the highest activity is(are) further fractionated and the new fractions are assayed. The bioassay-fractionation-bioassay sequence can go around until a promising candidate molecule or family of molecules are considered and then it is characterized. The first step of fractionation is typically a liquid–liquid extraction (solvent–solvent partition), and normally uses chromatography techniques, as TLC and column chromatography [201,202,203]. Isolated fractions are assayed afterwards. A final deep characterization using MS and NMR spectroscopy will reveal the exact structure of the bioactive molecule(s). This workflow will be a valuable tool to guide new drug discoveries and for understanding the structure-activity relationships. In this regard, lipidomics represents a valuable platform to advance in the characterization of lipid structures, survey lipid pathways, and can help in the elucidation of the interaction of lipids with microorganisms’ membranes, because of the high sensitivity and selectivity of MS.

## 6. Prospection and Applications of Antimicrobial Lipids

Several antimicrobial lipids are commercially available or can be used in different formulations in the cosmetic, pharmaceutical, and food industries, as well as in agriculture and aquaculture.

### 6.1. Cosmetic and Drug Formulations

In cosmetics, the free FA 10:0 and 12:0 and their corresponding MAG, monocaprin MAG(10:0) and monolaurin MAG(12:0), respectively, are commonly used in topical applications [21]. As a general rule, the esterification of free FA to glycerol increases the antimicrobial activity [127]. MAG(10:0) has been suggested as an ingredient for drug formulations to avoid oral infections by *C. albicans* [204].

### 6.2. Food Additives

The free FA 8:0 (commercial lipid standard) was shown to be effective against *E. coli*, *Salmonella* and other foodborne pathogens [21]. MAG(10:0) is known as a safe food additive, widely used as an emulsifier in the food industry [205].

Lipid extracts from marine organisms demonstrated to be a source of antibacterial compounds. The FA 16:1 inhibited the growth of the foodborne pathogens *Bacillus cereus* and *B. weihenstephanensis* [125]. Extracts from the microalga *D. salina* inhibited the growth of important food industry pathogens (i.e., *E. coli, S. aureus, C. albicans,* and *A. niger*) [123]. EPA, a high abundant PUFA in marine species, presented a potent action against the foodborne pathogen *B. cereus*, likely by disrupting the pathogen’s cell membrane, ultimately leading to cell lysis [206].

### 6.3. Herbicides and Pesticides

Some studies evaluated the antimicrobial activity of plants’ lipid extracts against both phytopathogenic fungi and bacteria. The sphingolipid [(2*S,*3*S,*4*R,*10*E*)-2-[(2’*R*)-2-hydroxytetracosanoylamino]-1,3,4-octadecanetriol-10-ene], a low polarity aglycone isolated from cucumber (*C. sativus*) stems, had strong antibacterial and antifungal activity against plant pathogens [74]. A mixture of lipids (FA, FAME, squalene, and *β*-sitosterol) extracted from andiroba (*Carapa* sp.) seeds demonstrated a MIC range of 156–250 µg/mL against several phytopathogenic fungi: *A. flavus*, *A. niger* and *F. oxysporum* [58]. Antimicrobial compounds from plants are envisioned as eco-friendly alternatives to chemical pesticides that harm both the environment and public health [58], because of their structural diversity, unique bioactivity, and environmental compatibility [74]. It is, thus, necessary to invest in a new approach to understand the mode of action of antimicrobial lipids isolated from plants in microbial cells.

Marine macroalgae have been traditionally used by several populations as soil fertilizers in agriculture, enriching them with minerals and plant growth promoters. The presence of antimicrobial molecules in macroalgae may contribute to develop biologically active compounds that protect agricultural crops toward pathogenic bacteria and fungi. This is the case of the sulfolipids isolated from the brown macroalga *S. wightii* that showed activity against the Gram-negative bacterium *Xanthomonas oryzae* pv. *oryzae* that causes the bacterial blight of rice [120]. Specific glycolipids, termed caminosides, isolated from the marine sponge *C. sphaeroconia* were active against the plant pathogen *Pythium ultimum*, the cause of damping off and root rot diseases in food crops and ornamental species [176]. Three sesterterpenes identified in the sponge *Thorectandra* sp. showed high inhibitory effect against the plant pathogenic fungus *Cladosporium herbarum* [190].

A recent study has mapped soil-borne fungal plant pathogens and projected their distribution under different climate change and soil uses scenarios [207]. The most dominant pathogens that harm global crop production belong to the genera *Alternaria* and *Fusarium* [207]. Enriched fractions of glycolipids from the macroalgae *L. cichorioides* and *S. pallidum*, as well as free FA and PUFA fractions from *L. cichorioides*, and FA esters, triacylgycerols/free sterols, free sterols and diacylglycerol fractions from *S. pallidum* demonstrated activity against the fungus *F. oxysporum* [91,118]. Lipid fractions with several steroid compounds from the oyster *S. glomerata* were very active against *Fusarium* sp. [171]. A bioprospection study in macroalgae of the order Caulerpales isolated ten sesquiterpenoids and diterpenoids and tested their activity in several bacterium and fungi strains. Eight of these compounds inhibited the growth of *Alternaria* sp. [98].

The free FA 10:0, 12:0, and their corresponding MAG [MAG(10:0) and MAG(12:0)], likewise as for cosmetic applications as mentioned above, are also used in agriculture crops as herbicides [21].

### 6.4. Aquaculture

Nowadays, aquaculture production accounts for almost half of the animal and aquatic plants catches, with 82 million tons [208]. Aquaculture represents an important food production sector, so finding environmental-friendly compounds with antibiotic activity instead of synthetic drugs is of utmost importance. This is even more urgent if we consider that, although vaccination is the best alternative to prevent infectious diseases in general, in aquaculture it is not likely to protect juvenile fish (without a mature immune system), shrimp, and bivalves (without an adaptive immune system, only with innate system), which are the most affected aquaculture animals by infectious diseases.

Several antimicrobial compounds identified in marine organisms are salt-tolerant [14] and have demonstrated to be efficient against aquaculture-relevant pathogens such as *Listonella anguillarum*, *Lactococcus garvieae,* and *Vibrio* species, showing advantage in pathogen control of fish and shellfish farming [99,108,112,125,126,209]. Extracts of *Falkenbergia*, a heteromorphic sporophyte of *Asparagopsis taxiformis*, inhibited the growth of several fish and shrimp *Vibrio* pathogens [106]. The FA 16:3 *n*-4 (HTA) isolated from the diatom *P. tricornutum* inhibited the growth of the Gram-negative marine pathogenic bacterium *L. anguillarum* [125]. A lipid fraction of the oyster *S. glomerata* extract inhibited the shrimp white spot syndrome virus (WSSV) in *Fenneropenaeus indicus* [171].

## 7. Conclusions

Lipids isolated from plants and marine organisms have demonstrated a broad spectrum of antimicrobial activities. These biomolecules can be envisaged as a promising alternative to help control pathogenic microbial infections. They have shown positive results in in vitro studies and microorganisms do not develop resistance to them, as far as it is known. However, the study of natural sources-derived antimicrobial lipids presents yet some shortcomings. There is a low number of systematic studies performing the isolation of pure lipid molecular species and their structural characterization, since they are mainly studied as complex extracts. There is also a lack of in vivo tests, indispensable to understand their mechanism of action, their cytotoxicity to eukaryotic cells, and consequently allowing their use at a large scale. Lipidomics is a fundamental tool to advance in the characterization of lipid structures and elucidate the lipids–microorganisms’ interaction. This can be achieved along with complementary biophysical, spectroscopic, and spectrometric studies that will be useful to understand their mechanism of action toward microbes and to define the structure–activity relationship. In addition, it is necessary to study the synergistic effects of these compounds against a wide range of microorganisms’ species to evidence broad-spectrum effects. The bioprospection of new natural lipids will benefit from the association among complementary research areas and the industry. Furthermore, the potential applications of antimicrobial lipids in different economic sectors, such as pharmaceutical, cosmetics, agriculture, or aquaculture, are still underexplored. So, the combination of these natural resources with sophisticated analytical tools applied to lipidomics research will open new perspectives on antimicrobial lipids for clinical and non-clinical applications. It is hoped that the future could bring about a greater interaction between lipidomics and natural chemotherapy to reveal the true microbicidal action of lipids toward pathogens. More studies must be done to understand which lipids can effectively be responsible by the inhibitory or microbicidal effect and their structure–activity relationship.

## Figures and Tables

**Table 1:** Plant potential antimicrobial lipids or lipid-rich extracts, their origin and extraction method grouped by botanic family.

Botanical Name	Family	Common Name	Country of Collection	Plant Part	Extracting Solvent/Method	Isolated Lipids or Lipid Mixtures	Ref.
*Sesuvium portulacastrum* L.	Aizoaceae	Sea purslane	India	Leaves	MeOH/benzene/sulfuric acid (200:100:10, *v*/*v*)	FAME	[60]
*Blutaparon portulacoides* (A. St.-Hil.) Mears	Amaranthaceae	Capotiraguá	Brazil	Roots	EtOH	Acyl steryl glycosides (sitosteryl 3-*β*-*O*-glucoside 6’-*O*-palmitate and stigmasteryl 3-*β*-*O*-glucoside 6’-*O*-palmitate)	[70]
*Arthrocnemum indicum* (Willd.) Moq*., Salicornia brachiata* Roxb.*, Suaeda maritima* (L.) Dumort. and *Suaeda monoica* Forsk.		Glasswort for *Salicornia* genus, herbaceous seepweed for *S. maritima*, and South-Indian seepweed for *S. monoica*	India	Shoots of *A. indicum* and *S. brachiata*, and leaves of *S. maritima* and *S. monoica*	Dry MeOH/benzene/sulfuric acid (200:100:10, *v*/*v*)	FAME	[61]
*Alternanthera brasiliana*		Brazilian joyweed	Brazil	Root, stem and leaves	EtOH and EtOAc	Linoleate oxylipins	[65]
*Phoenix dactylifera* L.	Arecaceae	Date palm	India	Seeds	CHCl_3_ and acetone	Sterol and triterpenes	[66]
*Asphodelus aestivus* Brot.	Asphodelaceae (formerly Liliaceae)	Summer asphodel	Turkey	Seeds	Petroleum ether with Soxhlet extractor	FA (C4:0, 6:0, 8:0, 10:0, 16:0, 18:0, 21:0, 24:0, 14:1, 15:1, 18:1n9t, 20:1, 24:1, 18:2, 18:2n6t, 18:2n6c, 20:2n6, 20:3n3, 22:6n3, and others unidentified)	[54]
*Artemisia incisa* Pamp.	Asteraceae		Pakistan	Roots	MeOH and recovered after elution on a SiO_2_ column with CH_2_Cl_2_/MeOH (9:1, *v*/*v*) following previous elution with *n*-hexane/EtOAc (5:4, *v*/*v*)	Artemceramide-B	[73]
*Pteranthus dichotomus* Forssk. (also known as *P. echinatus* Desf.)	Caryophyllaceae		Algerian Sahara	Aerial parts	MeOH/H_2_O (80:20, *v*/*v*). Aqueous phase extracted successively with petroleum ether, EtOAc and *n*-BuOH. EtOAc fraction contained the sterols and steryl glycoside. BuOH fraction contained the glyceroglycolipids and the cerebroside.	BuOH fraction contained the compounds: 1-*O*-palmitoyl-3-*O*-(6-sulfo-α-D-quinovopyranosyl)-glycerol, 1,2-di-*O*-palmitoyl-3-*O*-(6-sulfo-α-D-quinovopyranosyl)-glycerol and soya cerebroside I. EtOAc fraction contained the compounds: stigmat-7-en-3-ol, spinasterol, *β*-sitosterol and *β*-sitosterol-3-*O*-glycoside	[78]
*Cucumis sativus* L.	Cucurbitaceae	Cucumber	China	Stems	CHCl_3_ fraction of the crude methanolic extract	Sphingolipids [(2*S*,3*S*,4*R*,10*E*)-2-[(2’*R*)-2-hydroxytetracosanoylamino]-1,3,4-octadecanetriol-10-ene, 1-*O*-*β*-D-glucopyranosyl-(2*S*,3*S*,4*R*,10*E*)-2-[(2’*R*)-2-hydroxytetracosanoylamino]-1,3,4-octadecanetriol-10-ene and soya-cerebroside I]	[74]
*Excoecaria agallocha*	Euphorbiaceae	Blind-your-eye mangrove	India	Leaves	Dry MeOH, benzene and sulfuric acid (200:100:10, *v*/*v*)	FAME	[62]
*Albizia adianthifolia* (Schumach) and *Pterocarpus angolensis* (DC)	Fabaceae	Flat crown Albizia and African teak, respectively	Nigeria and Botswana, respectively	Heartwood of *A. adianthifolia* and stem bark of *P. angolensis*	*n*-hexane, CHCl_3_, MeOH, and 10% MeOH (aq)	*n*-hexadecanoic acid (palmitic acid); oleic acid; chondrillasterol; stigmasterol, 24S 5α-stigmast-7-en-3-ol; 9,12-octadecadienoic acid (*Z,Z*)-, methyl ester; *trans*-13-octadecanoic acid, methyl ester; tetradecanoic acid; hexadecanoic acid, methyl ester; octadecanoic acid	[79]
*Baphia massaiensis*		Jasmine pea	Botswana	Seeds	*n*-hexane/1-propanol (3:1, *v*/*v*) with Soxhlet extractor	Seed oil (total FA)	[80]
*Cassia tora* L. (or *Senna tora* L. Roxb.)		Sickle Senna	India	Leaves and stem	Petroleum ether with Soxhlet extractor	FA (the major were palmitic acid, linoleic acid, linolenic acid, margaric acid, melissic acid, and behenic acid)	[55]
*Trigonella foenum-graecum* L.		Fenugreek	India	Seeds	Supercritical fluid extraction (40–60 °C and 10–25 Mpa)	Conjugated linoleic acid methyl ester, saturated FAME, steroids	[63]
*Quercus leucotrichophora* A. Camus	Fagaceae	Banjh oak	India	Fruits	85% aqueous EtOH. Ethanolic extract fractionated with hexane and EtOAc using Soxhlet extractor. Hexane extract was analyzed.	FAME	[56]
*Quercus leucotrichophora* A. Camus		Banjh oak	Garhwal region of Himalaya	Leaves and bark	MeOH	FA; linoleic acid in stem bark and leaves extracts and *cis*-vaccenic acid in stem bark	[81]
*Vitex altissima* L., *V. negundo* L. and *V. trifolia* L.	Lamiaceae	Peacock chaste tree, Chinese chaste tree, and simpleleaf chastetree, respectively	India	Leaves	Dry MeOH/benzene/sulfuric acid (200:100:10, *v*/*v*)	FAME	[64]
*Linum usitatissimum* L.	Linaceae	Common flax or linseed	Algeria	Seeds	Petroleum ether with Soxhlet extractor	FAME	[57]
*Scaphium macropodum* (Miq.) Beumee ex. Heyne	Malvaceae	Malva nut or Kembang semangkok	Malaysia	Stem bark	MeOH	Methyl hexadecanoate, hexadecanoic acid <*n-*>	[82]
*Melastoma malabathricum* L.	Melastomataceae	Planter’s rhododendron or Sendudok	Malaysia	Leaves	MeOH/H_2_O (4:1, *v*/*v*), defatted with petroleum ether and extracted with CHCl_3_. Lipids recovered after elution of the CHCl_3_ extract by SiO_2_ column with CHCl_3_/acetone/MeOH (10:9:1, *v*/*v*).	Steryl glycoside: *β*-sitosterol 3-*O*-*β*-*D*-glucopyranoside	[69]
*Azadirachta indica* A. Juss	Meliaceae	Neem	India	Leaves	MeOH. Recovered after elution on a SiO_2_ column with CHCl_3_/MeOH (9:1, *v*/*v*)	SQDG	[71]
*Azadirachta indica* A. Juss		Neem	India	Leaves	Petroleum ether (60–80 °C) for 24 h and extracted thrice with MeOH for 48 h each time at room temperature	SQDG	[72]
*Carapa guianensis* Aubl. and *Carapa vasquezii* Kenfack		Andiroba	Brazil	Seed oil	*n*-hexane with Soxhlet extractor	FA, FAME, squalene, *β*-sitosterol	[58]
*Ficus lutea* Vahl	Moraceae	Giant-leaved fig or Lagos rubbertree	Cameroon	Woods	CH_2_Cl_2_/MeOH (1:1, *v*/*v*) and elution with EtOAc/10% MeOH	Glycosphingolipid [1-*O*-*β*-D-glucopyranosyl-(2*S*,3*R*,5*E*,12*E*)-2*N*-[(2′*R*)-hydroxyhexadecanoyl]-octadecasphinga-5,12-dienine] named lutaoside	[76]
*Ficus pandurata* Hance		Fiddle leaf fig	Egypt	Fruits	70% MeOH. MeOH extract fractionated on a SiO_2_ column and purified by semi-preparative HPLC to afford pure ceramides	Ceramides [panduramides A-D, and newbouldiamide]	[75]
*Kunzea ericoides* (A. Rich) J. Thompson	Myrtaceae	Kanuka (Maori), white manuka (Maori) or the white tea tree (English)	Australia	Leaves and twigs	CH_2_Cl_2_:MeOH (1:1, *v*/*v*), CH_2_Cl_2_:MeOH (2:1, *v*/*v*) and CH_2_Cl_2_ (neat)	Steryl esters, triacylglycerols, free FA, sterols, and phospholipids	[77]
*Pentagonia gigantifolia* Ducke	Rubiaceae		Peru	Roots	95% EtOH. EtOH extract was fractionated on a SiO_2_ column using CHCl_3_/MeOH from 0% to 100% MeOH. Fraction eluted with 2% MeOH/CHCl_3_ was separated on C18 SiO_2_ using 85% to 90% MeOH.	Acetylenic acids: 6-octadecynoic acid and 6-nonadecynoic acid	[38]
*Hedyotis pilulifera* (Pit.) T.N. Ninh			Vietnam	Aerial parts	MeOH at 60 °C, suspended in water and successively partitioned with CHCl_3_ and EtOAc. EtOAc extract fractionated on a SiO_2_ column.	Triterpenoids, steroids, FA, glycolipids, and a ceramide	[83]
*Withania somnifera* (L.) Dunal, *Euphorbia hirta* L., *Terminalia chebula* Retz.	Solanaceae, Euphorbiaceae, Combretaceae	Ashwaganda, asthma-plant, black myrobalan	India	Fruits, leaf, stem, and root from *W. somnifera* and *E. hirta* and fruits, leaf, stem, and stem bark from *T. chebula*	EtOAc	Sterols fraction	[67]
*Kaempferia pandurata* Roxb. (synonym of *Boesenbergia rotunda* (L.) Mansf.) and *Senna alata* (L.) Roxb.	Zingiberaceae and Fabaceae, respectively	Fingerroot and candle bush, respectively	Indonesia	Leaf of *S. alata* and rhizome of *K. pandurata*	EtOH (96%)	Sterols and triterpenoid	[68]
*Zygophyllum oxianum* Boriss.	Zygophyllaceae	Beancaper	Uzbekistan	Leaves, stems, and fruit	Acetone and CHCl_3_:MeOH (2:1, *v*/*v*) for total lipid extraction. Total lipids from each plant part separated in SiO_2_ columns. Neutral lipids eluted with CHCl_3_; glycolipids with acetone; phospholipids with MeOH.	Total lipid extract from leaves, stems and aerial organs (hydrocarbons, triterpenol and steryl esters, triacylglycerols, free FA, sterols, phospholipids)	[59]

**Table 2:** Antimicrobial activity of plant lipids or plant lipid-rich extracts.

Botanical Name	Tested (Micro)Organisms	Antimicrobial Testing Method/Evaluation	Reference Antimicrobial (Positive Control)	MIC, MBC, Diameter of Inhibition Zone (in mm) or Other	Isolated Lipids or Lipid Mixtures	Ref.
*Sesuvium portulacastrum* L.	G(+) bacteria: *Bacillus subtilis* NCIM 2063, *B. pumilus* NCIM 2327, *Micrococcus luteus* NCIM 2376 and *S. aureus* NCIM 2901; G(-) bacteria: *P. aeruginosa* NCIM 5031, *K. pneumoniae* NCIM 2957 and *E. coli* NCIM 2256. Ten isolates of MRSA and of MRSA NCTC 6571. Human pathogenic yeast type fungi: *Candida albicans*, *C. krusei*, *C. tropicalis* and *C. parapsilosis* and mould fungi: *Aspergillus niger*, *A. flavus,* and *A. fumigatus*	Inhibition zone (IZ) by disk diffusion test and minimum inhibitory concentration (MIC) by broth macrodilution method	Ciprofloxacin for bacteria, methicillin, oxacillin and vancomycin for MRSA and amphotericin-B for fungi	MIC: 0.25 mg/mL for *B. subtilis*, 0.5 mg/mL for *S. aureus*, MRSA, *P. aeruginosa*, *K. pneumoniae* and *C. albicans*, and 1.0 mg/mL for *E. coli*; MBC: 0.5 mg/mL for *B. subtilis*, 1.0 mg/mL for *S. aureus*, MRSA and *K. pneumoniae*, and 2.0 mg/mL for *P. aeruginosa* and *E. coli*; MFC: 1 mg/mL for *C. albicans*	FAME	[60]
*Blutaparon portulacoides* (A. St.-Hil.) Mears	*Trypanosoma cruzi*, *Leishmania amazonensis*, *S. aureus* ATCC 25923 and 7+ penicillinase producer, *Streptococcus epidermidis* (6ep), *E. coli* ATCC 10538, *Streptococcus mutans* (9.1), *Streptococcus sobrinus* (180.3)	Crude extracts and isolated compounds added to the trypomastigote-containing blood samples and incubated 24 h at 4 °C. Trypanocidal activity evaluated by counting the remaining trypomastigotes. *L. amazonensis* amastigote viability assessed colorimetrically by the reduction of a tetrazolium salt (MTT). Antimicrobial activity measured by the well-diffusionmethod in double layer	Gentian violet for trypanocidal activity and gentamicin for antibacterial assays	MIC: 100–500 μg/mL in *T. cruzi* trypomastigotes and 14–500 μg/mL in *L. amazonensis* amastigotes; 50 μg/mL in *E. coli*, *S. aureus* ATCC 25923, *S. aureus* (7+) and 500 μg/mL in *S. epidermidis*, *S. mutans,* and *S. sobrinus*	Acyl steryl glycosides (sitosteryl 3-*β*-*O*-glucoside 6’-*O*-palmitate and stigmasteryl 3-*β*-*O*-glucoside 6’-*O*-palmitate)	[70]
*Arthrocnemum indicum* (Willd.) Moq.*, Salicornia brachiata* Roxb*., Suaeda maritima* (L.) Dumort. *and Suaeda monoica* Forsk.	G(+) bacteria: *B. subtilis* NCIM 2063, *B. pumilus* NCIM 2327, *M. luteus* NCIM 2376, and *S. aureus* NCIM 2901; G(-) bacteria: *P. aeruginosa* NCIM 5031, *K. pneumoniae* NCIM 2957, and *E. coli* NCIM 2256; ten isolates of MRSA and of MRSA NCTC 6571; yeasts (*C. albicans, C. krusei, C. tropicalis,* and *C. parapsilosis)* and molds (*A. niger*, *A. flavus,* and *A. fumigatus*)	Disk diffusion method and broth macrodilution method	Ciprofloxacin for bacteria, methicillin, oxacillin and vancomycin for MRSA and amphotericin-B for fungi	MIC of 0.06 mg/mL of *S. brachiata* extracts against *B. subtilis*, *S. aureus,* and MRSA, and 0.5 mg/mL against *P. aeruginosa*; MIC of 0.5 mg/mL of all FAME extracts against *E. coli* and *K. pneumoniae*; MBC of 0.1 mg/mL of *S. brachiata* extracts against *P. aeruginosa* and of 1.0 mg/mL of all FAME extracts against *E. coli* and *K. pneumoniae*	FAME	[61]
*Alternanthera brasiliana*	*E. coli* ATCC 25922, *B. subtilis* ATCC 6623, *P. aeruginosa* ATCC 15442, *M. luteus* ATCC 9341, and *S. aureus* ATCC 25923	Microdilution broth method according to NCCLS standardization	Tetracycline and norfloxacin	MIC: 50 μg/mL against *B. subtilis*, *M. luteus,* and *S. aureus*	Linoleate oxylipins	[65]
*Phoenix dactylifera* L.	*Bacillus cereus* and *E. coli*	Disk diffusion method	Streptomycin	20 mm against *E. coli* and 17 mm against *B. cereus* at 1 mg/mL of the acetone extract	Sterol and triterpenes	[66]
*Asphodelus aestivus* Brot.	G(+) bacteria: *S. aureus* ATCC 6538-p, *E. faecalis* ATCC 29212; G(-) bacteria: *E. coli* ATCC 29998, *K. pneumoniae* ATCC 13883, *P. aeruginosa* ATCC 27853); yeasts: *C. albicans* ATCC 10239 and *C. krusei* ATCC 6258	Disk diffusion method and broth microdilution tests according to the recommendations of Clinical and Laboratory Standards Institute (CLSI)	Ampicillin, ciprofloxacin and fluconazole	MIC: 512 μg/mL against *S. aureus*, *E. faecalis*, *K. pneumoniae,* and *C. albicans*	FA (4:0, 6:0, 8:0, 10:0, 16:0, 18:0, 21:0, 24:0, 14:1, 15:1, 18:1n9t, 20:1, 24:1, 18:2, 18:2n6t, 18:2n6c, 20:2n6, 20:3n3, 22:6n3, and others unidentified)	[54]
*Artemisia incisa* Pamp.	*S. epidermidis* and *S. aureus*	Agar well diffusion method and MIC determined by a referenced method	Streptomycin and tetracycline	*S. epidermidis* (0.0157 mg/mL) and *S. aureus* (0.0313 mg/mL)	Artemceramide-B	[73]
*Pteranthus dichotomus* Forssk. (also known as *P. echinatus* Desf.)	*S. aureus* ATCC 25923, *E. coli* ATCC 25922, *K. pneumoniae* ESBL, and *Enterobacter sp.* ESBL	Disk diffusion method	Gentamicin and ampicillin	*P. dichotomus* BuOH extracts at 0.25 g/mL (8 mm against *E. coli*, *K. pneumoniae* ESBL); *P. dichotomus* EtOAc extract at 0.5 g/mL (7 mm against *E. coli*), at 65 mg/mL (8.33 mm against *S. aureus*), and at 0.25 g/mL (7 mm against *Enterobacter sp.* ESBL)	BuOH fraction contained 1-*O*-palmitoyl-3-*O*-(6-sulfo-α-D-quinovopyranosyl)-glycerol, 1,2-di-*O*-palmitoyl-3-*O*-(6-sulfo-α-D-quinovopyranosyl)-glycerol and soya cerebroside I. EtOAc fraction contained stigmat-7-en-3-ol, spinasterol, *β*-sitosterol and *β*-sitosterol-3-*O*-glucoside	[78]
*Cucumis sativus* L.	Phytopathogenic fungi (*Pythium aphanidermatum*, *Botryosphaeria dothidea*, *Fusarium oxysporum* f.sp. *cucumerinum,* and *Botrytis cinerea*); phytopathogenic bacteria [G(-): *Xanthomonas vesicatoria* ATCC 11633, *Pseudomonas lachrymans* ATCC 11921, and G(+) *B. subtilis* ATCC 11562]	Mycelial radial growth inhibition assay and antifungal activity (pour plating method in potato dextrose agar medium) for fungi and agar-well diffusion assay for bacteria	Carbendazim for fungi and streptomycin sulfate for bacteria	5.5–100 inhibitory rate of mycelia growth inhibitory activity; IC_50_ of *B. subtilis* (50.2–110.9 μg/mL), *X. vesicatoria* (25.6–64.5 μg/mL), *P. lachrymans* (15.3–37.3 μg/mL) for sphingolipids	Sphingolipids [(2*S*,3*S*,4*R*,10*E*)-2-[(2’*R*)-2-hydroxytetracosanoylamino]-1,3,4-octadecanetriol-10-ene, 1-*O*-*β*-D-glucopyranosyl-(2*S*,3*S*,4*R*,10*E*)-2-[(2’*R*)-2-hydroxytetracosanoylamino]-1,3,4-octadecanetriol-10-ene and soya-cerebroside I]	[74]
*Excoecaria agallocha*	G(+) bacteria: *B. subtilis* NCIM 2063, *B. pumilus* NCIM 2327, *M. luteus* NCIM 2376, *S. aureus* NCIM 2901; G(-) bacteria: *P. aeruginosa* NCIM 5031, *K. pneumoniae* NICM 2957, and *E. coli* NCIM 2256; yeasts: *C. albicans*, *C. krusei*, *C. tropicalis,* and *C. parapsilosis*	Disk diffusion method for antibacterial and antifungal susceptibility tests; MIC tested in Mueller-Hinton broth for bacteria and yeast nitrogen base for yeasts by two-fold serial dilution method	Ciprofloxacin and amphotericin B	MIC: 0.125 mg for *B. subtilis* and *S. aureus*, 0.5 mg for *P. aeruginosa* and *K. pneumoniae*, and 1.0 mg for *E. coli*; MBC: 0.25 mg for *B. subtilis* and *S. aureus*, 1.0 mg for *P. aeruginosa* and *K. pneumoniae*, and 2.0 mg for *E. coli*; MFC: 1 mg for *C. albicans*, *C. krusei* and *C. parapsilosis*	FAME	[62]
*Albizia adianthifolia* (Schumach) and *Pterocarpus angolensis* (DC)	Bacteria (*E. coli*, *P. aeruginosa*, *B. subtilis*, *S. aureus*) and yeast (*C. albicans*)	Modified agar overlay method	Chloramphenicol for bacteria and miconazole for fungi	MIQ: 1 μg of *n*-hexane and CHCl_3_ extracts of *A. adianthifolia* against *E. coli*; 50 μg of *n*-hexane and CHCl_3_ extracts of *A. adianthifolia* against *P. aeruginosa*; 50 μg of CHCl_3_ extract of *P. angolensis* against *B. subtilis* and 100 μg of *n*-hexane extract of *P. angolensis* against *B. subtilis* and *C. albicans*	*n*-hexadecanoic acid (palmitic acid); oleic acid; chondrillasterol; stigmasterol, 24S 5α-stigmast-7-en-3-ol; 9,12-octadecadienoic acid (*Z,Z*)-, methyl ester; *trans*-13-octadecanoic acid, methyl ester; tetradecanoic acid; hexadecanoic acid, methyl ester; octadecanoic acid	[79]
*Baphia massaiensis*	*E. coli*, *B. subtili*s*, P. aeruginosa*, *S. aureus,* and *C. albicans*	Agar well diffusion method	Not mentioned	10 mm of inhibition zone against *E. coli* and *S. aureus*, and 16 mm against *B. subtilis*	Seed oil (total FA)	[80]
*Cassia tora* L. (or *Senna tora* L. Roxb.)	MRSA, MSSA, *B. subtilis,* and *P. aeruginosa*	Broth microdilution method	Ampicillin	MIC for all bacteria between 125–1000 μg/mL	FA (the major were palmitic acid, linoleic acid, linolenic acid, margaric acid, melissic acid, and behenic acid)	[55]
*Trigonella foenum-graecum* L.	G(-) bacteria: *E. coli* and *P. aeruginosa*; G(+) bacteria: *S. aureus* and *Streptococcus pyogenes*; acid-fast bacteria: *M. tuberculosis*; fungi: *C. albicans*, *A. niger* and *A. clavatus*; parasite *Plasmodium falciparum* (etiological agent of malaria)	Antimicrobial activity assessed by broth dilution method, anti-tuberculosis activity assessed by the slope method, in vitro anti-malarial assay according to a microassay protocol	Gentamycin, chloramphenicol, ciprofloxacin and norfloxacin for bacteria; isoniazid and rifampicin for mycobacteria; nystatin and greseofulvin for fungi; chloroquine and quinine as anti-malarials	MIC values of 100, 250, 125 μg/mL towards *E. coli*, *S. aureus,* and *S. pyogenes* and *P. aeruginosa*, respectively. MFC value of 250 μg/mL of *C. albicans*. MIC value of 100 μg/mL toward *M. tuberculosis* and of 0.29 μg/mL toward *P. falciparum*	Conjugated linoleic acid methyl ester, saturated FAME, steroids	[63]
*Quercus leucotrichophora*, A. Camus	G(+) bacteria: *B. subtilis* and *S. aureus*; G(-) bacteria: *P. aeruginosa* and *E. coli*	Disk diffusion method for antibacterial susceptibility tests; MIC was tested in Mueller-Hinton broth for bacteria by two-fold serial dilution method	Ciprofloxacin	MIC: 0.125 mg/mL for *B. subtilis* and *S. aureus*; 0.5 mg/mL for *P. aeruginosa* and 1.0 mg/mL for *E. coli*	FAME	[56]
*Quercus leucotrichophora* A. Camus	G(-) bacteria: *E. coli* MTCC-582 and *P. aeruginosa* MTCC-2295; G(+) bacteria: *S. aureus* MTCC-3160, *B. subtilis* MTCC-441 and *S. pyogenes* MTCC-1924	Disk diffusion method	Ampicillin	IZ of both extracts against all microorganisms: 8.53 ± 0.50 to 19.07 ± 0.31 mm	FA; linoleic acid in stem bark and leaves extracts and *cis*-vaccenic acid in stem bark	[81]
*Vitex altissima* L., *V. negundo* L. and *V. trifolia* L.	*Culex quinquefasciatus* (early fourth-instar larvae)	Larvicidal activity analyzed according to standard procedures (WHO-VBC 81.807, 1981)	Not mentioned	*V. trifolia* (LC_50_ = 9.26 ppm and LC_90_ = 21.28 ppm)	FAME	[64]
*Linum usitatissimum* L.	*A. flavus* MTTC 2799 and *A. ochraceus* CECT 2092	Determination of percent mycelial inhibition by growth radial technique on solid medium and by biomass technique on liquid medium	Not mentioned	Antifungal index of FAME in solid medium: 54.19 ± 0.85 at 10 μL in *A. flavus* and 40.48 ± 0.12 at 90 μL for *A. ochraceus* at 90 μL	FAME	[57]
*Scaphium macropodum* (Miq.) Beumee ex. Heyne	*Mycobacterium smegmatis*, *E. coli*, *S. typhimurium*, *B. subtilis*, and *S. aureus*	Inhibitory activity of the extract by disk diffusion method; broth microdilution assay (MTT assay) was used to determine the MIC; MBC was determined via streak plate method	Ampicillin and rifampicin	IZ of 10.67 ± 0.58 mm in *S. aureus* and of 9 mm in *P. aeruginosa* at 0.25 mg/mL. *S. aureus* showed the lowest MIC (0.78 mg/mL) and MBC (3.13 mg/mL). For *M. smegmatis*, MIC value was 3.13 mg/mL and MBC was 25 mg/mL	Methyl hexadecanoate, hexadecanoic acid <*n-*>	[82]
*Melastoma malabathricum* L.	*S. aureus* ATCC 25923, *B. cereus* ATCC 10876, *P. aeruginosa* ATCC 17853, *S. typhi* laboratory strain	Disk diffusion method	Rifampicin	*P. aeruginosa* (9 mm at 0.25 mg/mL), *S. aureus* (7 mm, 1 mg/mL), *S. typhi* (9 mm at 1 mg/mL), *B. cereus* (10.5 mm at 2 mg/mL)	Steryl glycoside: *β*-sitosterol 3-*O*-*β*-D-glucopyranoside	[69]
*Azadirachta indica* A. Juss	Multidrug-resistant clinical isolates of *S. aureus*, *Salmonella enterica* serovar *typhi*, *S. dysenteriae*, *E. coli*, *Vibrio cholerae*, *K. pneumoniae,* and *P. aeruginosa*	MIC determined by microbroth dilution method and antibacterial sensitivity of SQDG determined by disk diffusion method (CLSI protocol)	Not mentioned	MIC of 32 μg/mL for *S. typhi* and two isolates of *S. dysenteriae*; MIC of 64 μg/mL for three isolates of *S. typhi*, *E. coli* and *V. cholerae* and 256 μg/mL for *K. pneumoniae*	SQDG	[71]
*Azadirachta indica* A. Juss	*Raillietina* spp. (helminth parasite)	Ultrastructural changes by scanning electron microscopy	Praziquantel	Anthelmintic activity of SQDG with 0.5 and 1 mg/mL, respectively: paralysis time of 1 h and 0.7 h; death time of 1.6 h and 0.9 h	SQDG	[72]
*Carapa guianensis* and *Carapa vasquezii*	Phytopathogenic fungi: *A. flavus*, *A. niger,* and *F. oxysporum*	Fungal mycelial growth inhibition trials developed in 96-well microtiter plates adding 10 μL of conidia suspensions (2 × 10^5^ conidia mL^−1^) and 90 μL yeast peptone dextrose. Inhibition of germination observed under light microscopy	20 mM hydrogen peroxide	MIC (μg/mL): 125–250 of *C. guianensis* and 15.6–125 of *C. vasquezii* against the three phytopathogenic fungi	FA, FAME, squalene, *β*-sitosterol	[58]
*Ficus lutea* Vahl	*Mucor miehei* and *B. subtilis*	Disk diffusion method	Nystatin	IZ of 17 mm for *M. miehei*; of 16 mm for *B. subtilis*; and of 12 mm for *C. albicans* exposed to 40 μg of compound	Glycosphingolipid [1-*O*-*β*-D-glucopyranosyl-(2*S*,3*R*,5*E*,12*E*)-2*N*-[(2′*R*)-hydroxyhexadecanoyl]-octadecasphinga-5,12-dienine] named lutaoside	[76]
*Ficus pandurata* Hance	Yeast: *C. albicans* ATCC 90028, *C. glabrata* ATCC 90030, *C. krusei* ATCC 6258, *A. fumigatus* ATCC 90906, MRSA ATCC 33591, *Cryptococcus neoformans* ATCC 90113, *S. aureus* ATCC 2921, *E. coli* ATCC 35218, *K. pneumoniae* ATCC 13883, *P. aeruginosa* ATCC 27853, and *Mycobacterium intracellulare* ATCC 23068); chloroquine sensitive (D6, Sierra Leone) and resistant (W2, Indochina) strains of *Plasmodium falciparum*; parasite: *Leishmania donovani* promastigotes	Modified versions of the NCCLS methods	Antibacterial agent and antifungal agents not mentioned; antimalarial agents: chloroquine and artemisinin; anti-leishmanial agents: pentamidine and amphotericin B	No activity was observed for any compound	Ceramides [panduramides A-D, and newbouldiamide]	[75]
*Kunzea ericoides* (A. Rich) J. Thompson	*E. coli* ATCC 25922 and *S. aureus* ATCC 25923	Broth microdilution method utilizing the redox dye resazurin	Not mentioned	0.625–10 mg/mL for *S. aureus* and more than 10 mg/mL in *E. coli*	Steryl esters, triacylglycerols, free FA, sterols and phospholipids	[77]
*Pentagonia gigantifolia* Ducke	*C. albicans* ATCC 90028 and fluconazole-resistant *C. albicans* strains	MIC and MFC determined by using a modified version of the microdilution NCCLS methods; sphingolipid reversal assay	Amphotericin B, fluconazole and flucytosine	*C. albicans* ATCC 90028 (0.52 to 1.04 μg/mL)	Acetylenic acids: 6-octadecynoic acid and 6-nonadecynoic acid	[38]
*Hedyotis pilulifera* (Pit.) T.N. Ninh	*S. aureus* NBRC 100910, *B. subtilis* NBRC 13719, *M. smegmatis* NBRC 13167	Microdilution method	Ampicillin	Oleanolic acid (MIC value of 2.5 μg/mL against *M. smegmatis*), rotungenic acid (MIC value of 2.5, 2.5, and 1.25 μg/mL against *S. aureus*, *B. subtilis*, and *M. smegmatis*, respectively), rotundic acid (MIC value of 5 μg/mL against *B. subtilis*)	Triterpenoids, steroids, FA, glycolipids, and a ceramide	[83]
*Withania somnifera* (L.) Dunal, *Euphorbia hirta* L., *Terminalia chebula* Retz.	*E. coli* MTCC 46, *P. aeruginosa* MTCC 1934), *Proteus mirabilis* MTCC 3310, *Raoultella planticola* MTCC 2271, *Enterobacter aerogenes* (now *Klebsiella aerogenes*) MTCC 2822, *B. subtilis* MTCC 121, *S. aureus* MTCC 3160	Disk diffusion method for antibiotic susceptibility testing. Broth microdilution method for determination of MIC values	Streptomycin	MIC: *W. somnifera* leaf (0.039 mg/mL on *P. aeruginosa*); *T. chebula* fruits and stems (0.039 mg/mL on *E. coli*) and *T. chebula* stems and fruits (0.039 mg/mL on *S. aureus*): MBC of 0.039 mg/mL of *T. chebula* bark on *S. aureus*	Sterols fraction	[67]
*Kaempferia pandurata* Roxb. (synonym of *Boesenbergia rotunda* (L.) Mansf.) and *Senna alata* (L.) Roxb.	MRSA, extended spectrum beta-lactamase (ESBL), and carbapenemase-resistant Enterobacteriaceae (CRE)	Broth microdilution method	Tetracycline and vancomycin for MRSA, cefotaxime and meropenem for ESBL-producing bacteria and for CRE	MIC: *K. pandurata* extract (256 μg/mL) and *S. alata* extract (512 μg/mL) against MRSA	Sterols and triterpenoid	[68]
*Zygophyllum oxianum* Boriss.	*S. aureus* ATCC 29213 and *B. subtilis* ATCC 6059	Modified disk diffusion method	Ampicillin, gentamicin sulfate, and nystatin	MIC: 2-mg leaves and stems extract (8 mm in *S. aureus* and 6 mm in *B. subtilis*, weak antibacterial activity); 2 mg-BuOH extract of whole air-dried aerial organs extract (5 mm in *B. subtilis*, 4 mm in *E. coli* and 20 mm in *C. maltosa,* good antifungal activity)	Total lipid extract from leaves, stems and aerial organs (hydrocarbons, triterpenol and steryl esters, triacylglycerols, free FA, sterols, phospholipids)	[59]

Abbreviations: CLSI, Clinical and Laboratory Standards Institute (formerly NCCLS); CRE, carbapenemase-resistant Enterobacteriaceae; ESBL, extended-spectrum beta-lactamase; FA, fatty acid; FAME, fatty acid methyl ester; G(-), Gram-negative; G(+), Gram-positive; IC_50_, half maximal inhibitory concentration; IZ: inhibition zone; LC_50_, concentration (ppm) at which 50% of larvae showed mortality; LC_90_, concentration (ppm) at which 90% of larvae showed mortality; MBC, minimum bactericidal concentration; MFC, minimum fungicidal concentration; MIC, minimum inhibitory concentration; MIQ, minimum inhibition quantity; MRSA, methicillin-resistant *Staphylococcus aureus*; MSSA, methicillin-sensitive *Staphylococcus aureus*; MTT, 3-(4,5-dimethylthiazol-2-yl)-2,5-diphenyltetrazolium bromide (tetrazolium dye); NCCLS, National Committee for Clinical Laboratory Standards; ppm, parts per million; SQDG, sulfoquinovosyldiacylglycerol.

**Table 3:** Algae lipids and lipid-rich extracts with antimicrobial potential, their origin and extraction method.

Scientific Name	Collection Site	Extracting Solvent(s)/Method	Isolated Lipids or Lipid Classes	Methods for Compounds Identification	Ref.
**Macroalgae–Chlorophyta**					
*Caulerpa racemosa*	Qionghai, Hainan, China	EtOH (95%). Extract partitioned with EtOAc and *n*-BuOH	SQDG [2S-1,2-di-*O*-palmitoyl-3-*O*-(6’sulfo-α-D-quinovopyranosyl) glycerol]	^1^H and ^13^C NMR, ESI-MS	[95]
*Caulerpa racemosa, Caulerpa lentillifera*	Port Dickson, Malaysia	CHCl_3_, MeOH	PUFA, MUFA, Terpenoids	LC-MS	[96]
*Caulerpa racemosa, Ulva fasciata*	Buzios, Rio de Janeiro, Brazil	Acetone insoluble material extracted with CHCl_3_/MeOH (2:1 and 1:2, *v*/*v*). Lipid extract partitioned on SiO_2_ column, eluted with CHCl_3_, acetone or MeOH	Glycolipid-rich extracts (Sulfoglycolipids, Glycosyldiacylglycerols)	HPTLC	[97]
*Caulerpa* spp., *Chlorodesmis fastigiata, Halimeda* spp.*, Penicillus capitatus, Penicillus dumentosus, Penicillus pyriformis, Rhipocephalus phoenix, Udotea argentea, Udotea cyathiformis, Udotea flabellum, Udotea petiolata*	Bahamas, Florida Keys, Puerto Rico, Belize, Guan, Hawaii, Australia, Mediterranean Sea	CH_2_Cl_2_. Chlorophylls removed with MgO_3_Si. Fractionation with SiO_2_ column and purification by HPLC	Sequiterpenoids, Diterpenoids	TLC, NMR, HPLC	[98]
*Chaetomorpha linum*	IMTA, Mar Piccolo of Taranto, Italy	CHCl_3_/MeOH (2:1, *v*/*v*), Soxhlet extractor, EtOH (95%)	Lipid extracts	^1^H and ^13^C NMR, 1D and 2D NMR, GC-FID, TLC	[99]
*Codium amplivesiculatum*	Bahía Magdalena, Mexico	CH_2_Cl_2_/EtOH (97:3, *v*/*v*). Liquid/liquid extraction CH_2_Cl_2_/H_2_O. Fractionation with CH_2_Cl_2_. Crystallization in hot MeOH	Fraction with clerosterol as main constituent. Isolated clerosterol did not show activity	^1^H NMR, IR	[100]
*Ulva fasciata*	Malvan, India	EtOH, fractionated by neutral alumina column with EtOAc/MeOH	Sphingosine (major component: *N*-palmitoyl-2-amino-1,3,4,5-tetrahydroxyoctadecane)	^1^H and ^13^C NMR, FAB-MS, IR	[101]
	Malvan, India	EtOH (90%). Extract fractionated. *n*-hexane fraction chromatographed on SiO_2_ and flash SiO_2_	Ceramide (Erythro-sphinga-4,8-dienine-*N*-palmitate)	^1^H and ^13^C NMR, ESI-MS, GC-MS, IR	[102]
	Mediterranean Sea, Egypt	CHCl_3_/MeOH (2:1, *v*/*v*). Glycolipid separation using acetone on SiO_2_ column	Glycolipid-rich extracts (DGDG)	GC-FID, LC-MS/MS	[103]
	Mediterranean Sea, Egypt	MeOH/CHCl_3_ (2:1, *v*/*v*). Sulfolipid isolation: diethylaminoethyl-cellulose column eluted with CHCl_3_/MeOH (6:4, *v*/*v*) and NH_3_	Sulfolipids (SQDG)	GC-MS, GC-FID, LC-MS/MS, IR	[89]
*Ulva rigida*	Cap Zebib and Ghar El Melh, Tunisia	CH_2_Cl_2_ and CH_2_Cl_2_/MeOH (1:1, *v*/*v*). Extracts fractionated on SiO_2_ column and TLC with *n*-hexane/EtOAc/CH_2_Cl_2_/MeOH	FA	^1^H and ^13^C NMR, GC	[27]
**Macroalgae–Rhodophyta**					
*Chondria armata*	Goa, West coast of India; Mumbai, India	MeOH and CHCl_3_, Polar fractions: petroleum ether/EtOAc (1:1, *v*/*v*), MeOH/CHCl_3_ (2:98, *v*/*v*), MeOH/CHCl_3_ (5:95, *v*/*v*)	Neutral glycolipids [main compound MGDG(20:5/16:0)]	^1^H and ^13^C NMR, ESI-MS/MS	[104]
*Chondrus crispus, Gracilaria vermiculophylla, Porphyra dioica*	IMTA and Portuguese coast, Portugal	EtOAc in Soxhlet extractor	FA	GC-FID	[105]
*Falkenbergia* (heteromorphic sporophyte of *Asparagopsis taxiformis*)	Kollam coast, India	MeOH. Fractionation on SiO_2_ column (petroleum ether/EtOAc and EtOAc/MeOH). Purification with TLC and RP HPLC	FA	GC-MS	[106]
*Galaxaura cylindrica, Laurencia papillosa*	Red Sea, Egypt	CHCl_3_/MeOH (2:1, *v*/*v*). Glycolipid separation using acetone on SiO_2_ columns	Glycolipid-rich extracts (DGDG)	LC-MS/MS	[103]
	Red Sea, Egypt	MeOH/CHCl_3_ (2:1, *v*/*v*). Sulfolipid isolation: diethylaminoethyl-cellulose column (CHCl_3_/MeOH (6:4, *v*/*v*) and NH_3_)	Sulfolipids (SQDG)	GC-MS, GC-FID, LC-MS/MS, IR	[89]
*Gigartina tenella*	Sagami Bay, Kanagawa, Japan	Acetone. Extract partitioned with EtOAc/H_2_O (3:1, *v*/*v*). Organic layer dissolved in EtOAc/MeOH/H_2_O (100:20:5, *v*/*v*) and chromatographed on SiO_2_ column	Glycolipid (Sulfolipids)	^1^H and ^13^C NMR, HR-FAB-MS	[107]
*Gracilaria gracilis*	Ganzirri lagoon and Margi channel, Eastern Sicily, Italy	CHCl_3_, Et_2_O in Soxhlet extractor	FA	GC-FID	[94]
*Gracilariopsis longissima*	Mar Piccolo of Taranto, Italy	CHCl_3_/MeOH/H_2_O (2:1:1, *v*/*v*)	FA	^1^H and ^13^C NMR, 1D and 2D NMR, GC-FID	[108]
*Hypnea musciformis, Osmundaria obtusiloba, Porphyra acanthophora, Pterocladiella capillacea*	Buzios, Rio de Janeiro, Brazil	Acetone insoluble material extracted with CHCl_3_/MeOH (2:1 and 1:2, *v*/*v*). Extract partitioned on SiO_2_ column (CHCl_3_, acetone or MeOH)	Glycolipid-rich extracts (Sulfolipids, Glycosyldiacylglycerols)	HPTLC	[97]
*Jania corniculata, Laurencia papillosa*	Suez Canal, Egypt	EtOH (70%), CH_2_Cl_2_	FA	GC-MS	[93]
*Laurencia okamurai*	Nanji Island in the East China Sea, Zhejiang Province, China	EtOH (95%) extract partitioned with Et_2_O and fractionated by SiO_2_ (gradient system: petroleum ether –CH_2_Cl_2_ (10:0 → 1:9)), Sephadex column, and purification by semi-preparative C18 HPLC	FA ethyl esters [(9*Z*,12*Z*,15*Z*,18*Z*,21*Z*)-ethyl tetracosa-9,12,15,18,21-Pentaenoate, (10*Z*,13*Z*)-ethyl nonadeca-10,13-dienoate, (9*Z*,12*Z*)-ethyl nonadeca-9,12-dienoate, (*Z*)-ethyl octadec-13-enoate (4), and (*Z*)-ethyl hexadec-11-enoate]	^1^H and ^13^C NMR, 1D and 2D NMR, IR, HR-EI-MS	[109]
*Laurencia* spp.	Pulau Tioman, Pahang, Pulau Karah, Terengganu, Pulau Nyireh, Terengganu, Malaysia	MeOH. Extract partitioned with Et_2_O and H_2_O and fractionated by SiO_2_ column (hexane/EtOAc)	Sesquiterpenes (Halogenated sesquiterpenes)	^1^H and ^13^C NMR, LREIMS, HREIMS	[110]
*Osmundaria obtusiloba*	Buzios, Rio de Janeiro, Brazil	Acetone insoluble material extracted with CHCl_3_/MeOH (2:1 and 1:2, *v*/*v*). Extract partitioned (CHCl_3_/MeOH/0.75% KCl (8:4:3, *v*/*v*)). Fractionation on SiO_2_ column (CHCl_3_, acetone and MeOH). MeOH fraction purified on SiO_2_ column (CHCl_3_/MeOH, 90:10, *v*/*v*)	Glycolipids (Sulfoglycolipids)	^1^H and ^13^C NMR, ESI-MS/MS	[111]
*Palmaria palmata, Grateloupia turuturu*	Batz-sur-Mer, France	CH_2_Cl_2_/MeOH (2:1, *v*/*v*), MeOH/H_2_O (1:1, *v*/*v*)	Polar lipids	^1^H and ^13^C NMR	[112]
*Pyropia orbicularis*	Maitencillo, Chile	MeOH, acetone, CH_2_Cl_2_, *n*-hexane. Soxhlet extractor. *n*-hexane extract fractionated on SiO_2_ column (2, 10, 20, 30 and 100% acetone)	Phospholipids (main compounds PC-O, PE, PS, PI, SM, GlCer), Glycolipids (MGDG), Triacylglycerol, DAG	LC-ESI-MS/MS	[113]
*Sphaerococcus coronopifolius*	Atlantic coast of Morocco	MeOH/CH_2_Cl_2_. Extract separated on SiO_2_ column (hexane, gradients of hexane/CH_2_Cl_2_ and CH_2_Cl_2_/acetone, and MeOH)	Bromoditerpenes (Sphaerolabdadiene-3,14-diol (1), Sphaerococcenol)	^1^H and ^13^C NMR, HRMS, EIMS, CIMS, FTIR, UV	[114]
**Macroalgae–Ochrophyta**					
*Dictyota cervicornis, Dictyota menstrualis*	Buzios, Rio de Janeiro, Brazil	Acetone insoluble material extracted with CHCl_3_/MeOH (2:1 and 1:2, *v*/*v*). Extract partitioned on SiO_2_ column (CHCl_3_, acetone or MeOH)	Glycolipid-rich extracts (Sulfoglycolipids, Glycosyldiacylglycerols)	HPTLC	[97]
*Dictyota fasciola, Taonia atomaria*	Mediterranean Sea, Egypt	CHCl_3_/MeOH (2:1, *v*/*v*). Glycolipid separation using acetone on SiO_2_ column	Glycolipid-rich extracts (DGDG)	GC-FID, LC-MS/MS	[103]
	Mediterranean Sea, Egypt	MeOH/CHCl_3_ (2:1, *v*/*v*). Sulfolipid isolation: diethylaminoethyl-cellulose column (CHCl_3_/MeOH (6:4, *v*/*v*) and NH_3_)	Glycolipids (Sulfolipids)	GC-MS, GC-FID, LC-MS/MS, IR	[89]
*Fucus evanescens*	West coast of Ungava Bay, Canada	EtOAc (99%), CH_2_Cl_2._ EtOAc algal extract acetylated and organic layer purified by flash chromatography (0% → 50% EtOAc in hexane and flushed with 100% EtOAc and 5% MeOH in 95% EtOAc)	Glycolipid-rich extracts	^1^H and ^13^C NMR	[115]
*Himanthalia elongata*	Las Palmas, Spain	Pressurized liquid extraction Hexane, EtOH, H_2_O	Sterol (Fucosterol), FA	GC-MS, HPLC-DAD	[116]
*Laminaria cichorioides*	Khasan region of the Primorskii Territory, in the Troitsa Gulf (the Sea of Japan), Russia	EtOH (96%). Lipophilic fraction extracted with CHCl_3_. Fractionation of lipid classes on SiO_2_ column	Glycolipids (MGDG, DGDG, SQDG), Free FA, PUFA		[91]
*Sargassum dentifolium*	Suez Canal, Egypt	EtOH (70%), CH_2_Cl_2_	FA	GC-MS	[93]
*Sargassum fusiforme, Sargassum vulgare*	Red Sea, Egypt	Et_2_O, MeOH, EtOH, CHCl_3_	Terpenoids, FA	GC-MS	[117]
*Sargassum pallidum*	Trinity Bay in the Peter the Great Gulf, Russia	EtOH; EtOH/acetone (1:1, *v*/*v*), EtOH/CHCl_3_ (1:1, *v*/*v*). Fractionation of lipid classes on SiO_2_ column (hexane, Et_2_O/hexane with increasing ether concentration (95:5 → 50:50, *v*/*v*) and CHCl_3_)	Glycolipids (MGDG, SQDG; DGDG), Free FA/Esters, Triacylglycerols, DAG		[118]
*Sargassum vulgare*	Sepetiba Bay, Brazil	CHCl_3_/MeOH (2:1 and 1:2, *v*/*v*). Fractionated on SiO_2_ column (CHCl_3_, acetone and MeOH)	Glycolipid-rich extracts (Sulfoglycolipids)	^1^H and ^13^C NMR, ESI-MS-MS	[119]
*Sargassum wightii*	Gulf of Mannar, India	MeOH. Isolation on SiO_2_ column (hexane/EtOAc, EtOAc/MeOH). Purification on flash SiO_2_ column (CHCl_3_/MeOH gradients)	Glycolipid (Sulfoglycerolipid, 1-*O*-palmitoyl-3-*O*(6′-sulfo-α-quinovopyranosyl)-glycerol)	^1^H and ^13^C NMR, IR	[120]
**Microalgae**					
*Chaetoceros muelleri*		Supercritical fluid extraction, EtOH (99.5%)	Triacylglycerol, DAG, MAG, sterols (cholesterol), FA	HPLC-ELSD, GC-FID	[121]
*Chlorococcum* HS-101	Japan	MeOH extract, partitioned with hexane. SiO_2_ column (MeOH/CHCl_3_ gradient). Active fraction recovered with MeOH/CHCl_3_ (5:95, *v*/*v*)	FA	^1^H and ^13^C NMR, GC-MS	[122]
*Dunaliella salina*	Jerusalem, Israel	Pressurized liquid extracts: hexane, petroleum ether, EtOH	FA Sesquiterpenoids (Neophytadiene), Diterpenoid (Phytol)	GC-MS	[123]
*Navicula delognei*	Lepreau Ledges, New Brunswick, Canada	MeOH, CHCl_3_, extract chromatographed on SiO_2_ column (CHCl_3_ and CHCl_3/_MeOH)	FA [(6*Z*,9*Z*,12*Z*,15*Z*)-hexadecatetraenoic acid, (6*Z*,9*Z*,12*Z*,15*Z*)-octadecatetraenoic acid), Ester ((E)-phytol (5*Z*,8*Z*,11*Z*,14*Z*,17*Z*)-eicosapentaenoate]	^1^H and ^13^C NMR, GC-MS	[124]
*Phaeodactylum tricornutum*	Experimental Phycology and Culture Collection of Algae at the University of Göttingen (Germany)	EtOAc and MeOH extracts applied to SiO_2_ Sep Pak cartridges. EtOAc extract eluted with 10% step increases of hexane/EtOAc until 100% EtOAc. MeOH extract eluted with 10% step increases of EtOAc/MeOH until 100% MeOH	Free FA (palmitoleic acid, HTA)	^1^H and ^13^C NMR, ESI-MS	[125]
		MeOH/H_2_O (5:1, *v*/*v*). Extract redissolved in MeOH (70%) and fractioned on Sep Pak cartridge (MeOH (70%) followed by constant volumes of MeOH (5% steps → 100%)). Fractionated by RP HPLC	FA (EPA)	^1^H NMR, ESI-MS	[126]
*Synechocystis* sp.	Las Palmas, Spain	Pressurized liquid extraction: hexane, EtOH, H_2_O	FA, Sesquiterpenoids (Neophytadiene)	GC-MS, HPLC-DAD	[116]

**Table 4:** Antimicrobial activity of algae lipids or algae lipid-rich extracts.

Scientific Name	Antimicrobial Activity	Tested Microorganisms	Antimicrobial Testing Method/Evaluation	Reference Antimicrobial (Positive Control)	MIC, MBC, Diameter of Inhibition Zone (IZ, in mm) or Other	Ref.
**Macroalgae–Chlorophyta**						
*Caulerpa racemosa*	Antiviral	Viruses: Cox B3, HSV	Cytopathic effect (CPE) reduction assay, 3-(4,5-dimethylthiazol-2-yl)-2,5-diphenyltetrazolium bromide (MTT) method	Acyclovir (HSV), Ribavirin (Cox B3)	IC_50_/CC_50_ (µg/mL)/SI Cox B3: 31.3/500/16 HSV: 7.9/250	[95]
*Caulerpa racemosa, Caulerpa lentillifera*	Antibacterial	G(+):MRSA (MTCC 381123) G(-): *E. coli* K1 (MTCC 710859)	Disk diffusion method, crude extracts (CHCl_3_ and MeOH)	Penicillin-streptomycin	PI: 97.7% (*C. racemosa)*	[96]
*Caulerpa racemosa, Ulva fasciata*	Antiviral	Viruses: HSV-1-ACVs, HSV-1-ACVr	Titer reduction			[97]
*Caulerpa* spp., *Chlorodesmis fastigiata, Halimeda* spp.*, Penicillus capitatus, Penicillus dumentosus, Penicillus pyriformis, Rhipocephalus phoenix, Udotea argentea, Udotea cyathiformis, Udotea flabellum, Udotea petiolata*	Antibacterial, Antifungal	G(-):*Serratia marinoruba, Vibrio splendida, V. harveyi, V. leiognathi, Vibrio* sp. Undescribed bacteria: VJP Cal8101, VJP Cal8102, VJP Cal8103 Fungi: *Leptosphaeria* sp. *Lulworthia* sp., *Alternaria* sp., *Dreschleria haloides, Lindra thallasiae*, Undescribed fungi: VJP Cal8104, VJP Cal8105	Plate assay-disk method		IZ (mm) > 2	[98]
*Chaetomorpha linum*	Antibacterial	G(+): *Pseudomonas* sp., *Staphylococcus* sp., *Streptococcus agalactiae*, *Enterococcus* sp. G(-): *Vibrio alginolyticus, V. harveyi, V. mediterranei, V. ordalii, V. parahaemolyticus, V. salmonicida, V. vulnificus* Yeast: *C. albicans*, *Candida famata, C. glabrata*	Disk diffusion method		IZ (mm) *V. ordalii*: 8–12 *V. vulnificus*: 8–12	[99]
*Codium amplivesiculatum*	Antibacterial	G(+): *S. aureus* (ATCC BAA-42, resistant to methicillin, penicillin, ampicillin/sulbactam, oxacillin, cefalotine) G(-): *V. parahaemolyticus* (17802)	Disk diffusion method		MIC (µg/mL)/IZ (mm) *S. aureus:* 125/15 *V. parahaemolyticus:* >250/8	[100]
*Ulva fasciata*	Antiviral	Virus: Semeliki Forest Virus (SFV)			20 mg/mouse/7 days by giving 50% protection	[101]
	Antiviral	Viruses: Japanese encephalitis virus (JEV), encephalomyocarditis (EMC) virus	96-well microtiter plates		CC_50_ (µg/mL)/EC_50_ (µg/mL)/TI JEV: 7.8/3.9/2.0	[102]
	Antifungal, Antiviral	Yeast: *C. albicans;* Fungus: *A. niger* Virus: HSV-1	Disk diffusion method; plaque reduction assay (antiviral)		MIC (µg/mL)/IZ (mm) *C. albicans:* 60/8, *A. niger*: 80/13 HSV-1: 9.37–15.62%	[103]
	Antibacterial, Antiviral	G(+):*B. subtilis* RRL B-94 G(-): *E. coli* NRRL B-3703 Virus: HSV-1	Disk diffusion method	Chloramphenicol (bacteria), Acyclovir (virus)	MIC (µg/mL)/IZ (mm) PI(%) *E. coli*: 60/13 *B. subtilis*: 40/16 HSV-1: 18.75–46.87%	[89]
*Ulva rigida*	Antibacterial	G(+): *S. agalactiae, S. aureus* (ATCC 25923), *S. aureus* (ATCC 6538), *E. faecalis* (ATCC 29212), *Micrococcus* sp. G(-): *Vibrio tapetis* (CECT4600), *V. anguillarum* (ATCC 12964T), *V. alginolyticus* (ATCC 17749T), *E. coli* O126-B16 (ATTC 14948), *E. coli* (ATCC 25922), *E. coli* (ATCC 8739), *Pseudomonas cepacia, P. fluorescens* (AH2), *P. aeruginosa* (ATCC 27853), *Aeromonas salmonicida* (LMG3780), *A. hydrophila* B3, *S. typhimurium* (C52) Yeast: *C. albicans* (ATCC10231)	Disk diffusion method and broth microdilution technique		IZ (mm) 6.3–16.3	[27]
**Macroalgae–Rhodophyta**						
*Chondria armata*	Antibacterial, Antifungal	G(+): *S. aureus* G(-): *E. coli, P. aeruginosa, S. typhi, Salmonella flexneri, Klebsiella* sp., *V. cholerae* Yeast: *C. albicans, C. neoformans, Rhodotorula* sp. Fungi: *Aspergillus fumigatus, A. niger*	Disk diffusion method	Streptomycin, Nystatin	1 < IZ ≤ 4	[104]
*Chondrus crispus, Gracilaria vermiculophylla, Porphyra dioica*	Antibacterial, Antifungal	G(+): *Listeria innocua* (NCTC 11286), *B. cereus* (ATCC 11778), *E. faecalis* (LMG S 19456 5002), *Lactobacillus brevis* (LMG 6906), *S. aureus* (ATCC 6538), MRSA G(-): *E. coli* (ATCC 8739), *Salmonella enteritidis* (ATCC 3076), *P. aeruginosa* (ATTC 10145) Yeast: *Candida* spp. (CCUG 49242)	Disk diffusion method	Ampicillin (*L. innocua*), Cycloheximide (*Candida* spp.), Chloramphenicol (other microorganisms)	IZ (mm) *C. crispus: 5* < IZ ≤ 20 *G. vermiculophylla:* 10 < IZ ≤ 15 *P. dioica:* 5 < IZ ≤ 12	[105]
*Falkenbergia* (heteromorphic sporophyte of *Asparagopsis taxiformis*)	Antibacterial	G(+): *S. epidermidis, S. aureus, B. subtilis* G(-): *V. vulnificus* (MTCC 1145)*, V. parahaemolyticus* (MTCC 451), *V. harveyi* (MTCC 3438), *V. alginolyticus* (MTCC 4439), *V. alcaligenes* (MTCC 4442), *P. aeruginosa, K. pneumoniae*	Broth dilution method	Chloramphenicol, Nalidixic acid	IZ (mm)/MIC (µg)/MBC (µg) *S. epidermidis*: 21/1250/270 *S. aureus*: 21/750/170 *B. subtilis*: 23/750/180 *V. vulnificus*: 31/750/90 *V. parahaemolyticus:* 28/750/110 *V. harveyi*: 26/750/60 *V. alginolyticus*: 32/500/80 *V. alcaligenes*: 33/500/50 *P. aeruginosa*: 19/1250/420 *K. pneumoniae*: 15/1250/380	[106]
*Galaxaura cylindrica, Laurencia papillosa*	Antiviral	Virus: HSV-1	Plaque reduction assay	*Acyclovir*	Inhibition (%) *L. papillosa*: 9.37–31.25 *G. cylindrica*: 15.62–28.12	[103]
	Antibacterial, Antiviral	G(+): *B. subtilis NRRL B-94* G(-): *E. coli NRRL B-3703* Virus: (HSV-1)	Disk diffusion method	Chloramphenicol (bacteria), Acyclovir (virus)	MIC (µg/mL)/IZ (mm) PI(%) *L. papillosa* *E. coli*: -/8 *B. subtilis*: -/11 HSV-1: 40.62–59.37% *G. cylindrica* *E. coli*: 80/11 *B. subtilis*: 80/11 HSV-1: 45.87–59.37%	[89]
*Gigartina tenella*	Antiviral	HIV-reverse transcriptase type 1			IC_50_ 11.2 µM	[107]
*Gracilaria gracilis*	Antibacterial	G(+): *B. subtilis* G(-): *V. fischeri, V. cholerae, P. aeruginosa*, *Salmonella* sp., *A. hydrophila*	Disk diffusion method	Chloramphenicol	MIC: 5 µg/disk IZ (mm) *B. subtilis* 10.3–17.6 (CHCl_3_)/10–15.6 (Et_2_O)	[94]
*Gracilariopsis longissima*	Antibacterial, Antifungal	G(+): *S. agalactiae, Enterococcus* sp. G(-): *P. aeruginosa*, *V. salmonicida*, *V. fluvialis, V. vulnificus, V. cholerae* non-O1, *V. alginolyticus* Yeast: *C. albicans, C. famata*, *C. glabrata*	Disk diffusion method		IZ (mm) *V. alginolyticus*: 25 *V. fluvialis*: 8 *V. vulnificus*: 15 *V. cholerae* non-O-1:10	[108]
*Hypnea musciformis, Osmundaria obtusiloba, Porphyra acanthophora, Pterocladiella capillacea*	Antiviral	Virus: HSV-1-ACVs, HSV-1-ACVr	Titer reduction		PI(%)/VII *O. obtusiloba* ACVs-HSV-1: 82.2–99.5/0.75–2.35 HSV-1-ACVr: 99.7–99.9/2.5–4.5	[97]
*Jania corniculata, Laurencia papillosa*	Antibacterial, Antifungal	G(+): *B. subtilis, Staphylococcus albus, E. faecalis* G(-): *E. coli* Yeast: *C. albicans* Fungus: *A. flavus*	Disk diffusion method		11 < IZ ≤ 15 No IZ (*A. flavus*)	[93]
*Laurencia okamurai*	Antifungal	Yeast: *C.* *neoformans* (32609)*, C. glabrata* (537) Fungi: *Trichophyton rubrum* (Cmccftla)*, A. fumigatus* (07544)	Broth dilution method	Amphotericin B, fluconazole, voriconazole, ketoconazole	MIC_80_ (µg/mL) *C. neoformans*: 8–64 *C. glabrata*: 4–64 *A. fumigatus*: >64 *T. rubrum*: 64	[109]
*Laurencia* spp.	Antibacterial	G(-): *Chromobacterium violaceum, P. mirabilis,* *P. vulgaris, Erwinia* sp., *V. parahaemolyticus, V. alginolyticus*	Disk diffusion method		MIC (µg/disk) *C. violaceum*: 10–40 *P. mirabilis*: 20–40 *P. vulgaris*: 20–40 *Erwinia* sp.: 10–30 *V. parahaemolyticus*: 20–40 *V. alginolyticus*: 20–30	[110]
*Osmundaria obtusiloba*	Antiviral	Virus: HSV-1, HSV-2	Titer reduction	Acyclovir	EC_50_ (µg/mL)/SI/PI(%) HSV-1: 42/1.7/75 HSV-2: 12/6/96	[111]
*Palmaria palmata, Grateloupia turuturu*	Antibacterial	G(-): *V. harveyi* ORM4	Broth microdilution method		0.2 < PI ≤ 7.9%	[112]
*Pyropia orbicularis*	Antibacterial	G( + ): *S. aureus, B. cereus* G(-): *E. coli*	Disk diffusion method	Kanamycin	16 < IZ < 26	[113]
*Sphaerococcus coronopifolius*	Antibacterial, Antiplasmodial	G(+): *S. aureus* (ATCC # 6538) Parasitic protozoa: *P. falsciparum* (FCB1)	Antibacterial: Disk-diffusion, Antimalarial: inhibition of [^3^H]-hypoxanthine uptake by *P. falsciparum* cultured in human blood		*S. aureus*-MIC: 0.104–0–146 µM *P. falciparum*-IC_50_: 1 µM	[114]
**Macroalgae–Ochrophyta**						
*Dictyota cervicornis, Dictyota menstrualis*	Antiviral	Virus: HSV-1-ACVs, HSV-1-ACVr	Titer reduction			[97]
*Dictyota fasciola, Taonia atomaria*	Antibacterial, Antifungal, Antiviral	G(+): *B. subtilis* G(-): *E. coli* Fungi: *C. albicans, A. niger* Virus: HSV-1	Disk diffusion method (antibacterial); Plaque reduction assay (antiviral)		*D. fasciola:* Inhibition (%) HSV-1: 50.00–81.25% *T. atomaria:* MIC (µm/mL)/IZ(mm) *B. subtilis*: 80/9*, E. coli:* 80/7, *C. albicans*: 80/10, *A. niger*: 60/12 Inhibition (%) HSV-1: 31.25–34.37%	[103]
	Antibacterial, Antiviral	G(+):*B. subtilis NRRL B-94* G(-): *E. coli NRRL B-3703* Virus: HSV-1	Disk diffusion method (antibacterial); Plaque reduction assay (antiviral)	Chloramphenicol (bacteria), Acyclovir (virus)	MIC (µg/mL)/IZ (mm) PI(%) *D. fasciola* *E. coli*: -/8 *B. subtilis*: -/10 HSV-1: 46.87–70.12% *T. atomaria:* *E. coli*: 60/15 *B. subtilis*: 40/13 HSV-1: 43.75–56.25%	[89]
*Fucus evanescens*	Antibacterial	G(+): *B. cereus, Clostridium difficile,* MRSA*, Propionibacterium acnes* (ATCC and clinical isolate), *S. pyogenes* G(-): *Acinetobacter baumannii, E. coli, Haemophilus influenzae, K. pneumoniae, Legionella pneumophila, P. aeruginosa*	Disk diffusion method		MIC_100_: 50 µg/mL	[115]
*Himanthalia elongata*	Antibacterial	G(+): *S. aureus* ATCC 25923, G(-): *E. coli* ATCC 11775 Yeast: *C. albicans* ATCC 60193 Fungi: *A. niger* ATCC 16404	Broth microdilution method		MBC (mg/mL) *S. aureus:* 6.25 *E. coli:* 6.00 MFC (mg/mL) *C. albicans:* 8 *A. niger:* 12	[116]
*Laminaria cichorioides*	Antibacterial, Antifungal	G(+): *S. aureus* ATCC 21027 G(-): *E. coli* ATCC 15034 Yeast: *Safale* S04, *C. albican*s KMM 455 Fungi: *A. niger* KMM 4634, *F. oxysporum* KMM 4639	Disk diffusion method	Fucoxanthin, Nitrofungin	IZ (mm) *S. aureus*: 2–5 *E. coli:*1–6 *C. albican*s: 1–6 A. *niger*: 1–3 *F. oxysporum*: 1–4	[91]
*Sargassum dentifolium*	Antibacterial, Antifungal	G(+): *B. subtilis, S. albus, E. faecalis* G(-): *E. coli* Yeast: *C. albicans* Fungus: *A. flavus*	Disk diffusion method		11 < IZ (mm) ≤ 12 No IZ (*A. flavus*)	[93]
*Sargassum fusiforme, Sargassum vulgare*	Antibacterial	Multidrug resistant: *S. aureus,* *P. aeruginosa*, *Shigella flexneri,* *E. coli,* *Corynebacterium* sp.	Agar well diffusion		9.33 < IZ (mm) ≤ 23.33 MIC: 50–100 mg/mL	[117]
*Sargassum pallidum*	Antibacterial, Antifungal	G(+): *S. aureus* ATCC 21027 G(-): *E. coli* ATCC 15034 Yeast: *C. albican*s KMM 455 Fungi: *A. niger* KMM 4634, *F. oxysporum* KMM 4639, *Septoria glycines*	Agar well diffusion		IZ (mm) *S. aureus*: 0.7–14.5 *E. coli*: 0.5–6.7 *C. albicans*: 1.0–4.5 *A. niger*: 2.0–5.7 *F. oxysporum*: 1.0–5.2 *S. glycines*: 2.0–5.7	[118]
*Sargassum vulgare*	Antiviral	Virus: HSV-1, HSV-2	Titer reduction	Acyclovir	PI (%) HSV-1: 96.0–99.9 HSV-2: 99.9	[119]
*Sargassum wightii*	Antibacterial	*Xanthomonas oryzae* pv. *oryzae* CAS ar01	Disk diffusion method		IZ (mm): 3.0–13.5	[120]
**Microalgae**						
*Chaetoceros muelleri*	Antibacterial, Antifungal	G(+): *Staphyloccocus aureus* (ATCC 25923) G(-):*E. coli* (ATCC 11775) Yeast: *C. albicans* (ATCC 60193)	Broth microdilution method	Chloramphenicol, Amphotericin B	MBC (mg/mL) *E. coli*: 12–15 *S. aureus*: 12–17 *C. albicans*: 7–9	[121]
*Chlorococcum HS-101*	Antibacterial	G(+): MRSA*, S. aureus* ATCC 25923	Disk diffusion method	Gentamicin, Amikacin, Cephalosporin, Habekacin, Ampicillin, Vancomycin, Oxytetracyclin, Erythromycin, Cefmetazole, Fosfomycin, Imipenem, Minomycin	IZ (mm): 18.7–28.3	[122]
*Dunaliella salina*	Antibacterial, Antifungal	G(+): *S. aureus* ATCC 25923 G(-): *E. coli* ATCC 11775 Yeast: *C. albicans* ATCC 60193 Fungi: *A. niger* ATCC 16404	Disk diffusion method	Chloramphenicol (bacteria), Amphotericin B (yeast and fungi)	MBC (mg/mL) *E. coli*: 6–30 *S. aureus*: 8–30 MFC (mg/mL) *C. albicans*: 12–30 *A. niger*: 32–>35	[123]
*Navicula delognei* f. *elliptica*	Antibacterial	G(+): *Staphyloccus aureus* (ATCC 25923)*, S. epidermidis* (ATCC 12228) G(-): *S. typhimurium* (ATCC 14028)*, P. vulgaris* (ATCC 13315)*, Enterobacter cloacae* (ATCC 23355)*, E. coli* (ATCC 25922), *K. pneumoniae* (ATCC 13883)*, Serratia marcescens* (ATCC 8100)	Disk diffusion method	Ampicillin, Tetracycline, Chloramphenicol	IZ (mm) *S. aureus* >4 *S. typhimurium* >4 *S. epidermidis* >2 *P. vulgaris* >2 *E. coli* IZ noticeable No IZ (*E. cloacae, K. pneumoniae, S. marcescens)*	[124]
*Phaeodactylum tricornutum*	Antibacterial, Antifungal	G(+): *S. aureus* (SH1000), *Bacillus weihenstephanensis* (10390), MRSA 252, MRSA 16a, *S.* *epidermidis, M. luteus* (NCIMB 9278), *Planococcus citreus* (NCIMB 1493), *B. cereus* (883-00) G(-): *Alteromonas* *haloplanktis* (NCIMB 19), *A. hydrophila* (NCIMB 1108), *Photobacterium* *phosphoreum* (NCIMB 64), *Psychrobacter immobilis* (NCIMB 308), *Listonella anguillarum* (MT1637), *E. coli* B, *P. aeruginosa* (NCIMB 10775) Yeast: *C. glabrata, Candida neoformis,* *Candida sp*., *Saccharomyces cerevisiae* BY4741a	Disk diffusion method	Ampicillin	IC_50_ (µM)/MBC (µM) *S. aureus:* 10–40/40–80	[125]
	Antibacterial, Antifungal	G(+):*M. luteus* (NCIMB 9278), *Planococcus* *citreus* (NCIMB 1493), *B. cereus* (883-00), *S. aureus* (SH1000), *B. weihenstephanensis* (10390), MRSA 252, MRSA16a, *S. epidermidis* G(-): *Alteromonas haloplanktis* (NCIMB 19), *A. hydrophila* (NCIMB 1108), *Photobacterium phosphoreum* (NCIMB 64), *Psychrobacter immobilis* (NCIMB308), *Listonella anguillarum* (MT1637), *E. coli* B, *P. aeruginosa* (NCIMB 10775) Yeast:*C. glabrata, C. neoformis, Candida* sp., *S. cerevisiae* BY4741a	Agar well diffusion		Growth inhibition (mm^2^) ≤50–>50 *S. aureus*: 25–190 mm^2^	[126]
*Synechocystis* sp.	Antibacterial	G(+): *S. aureus* ATCC 25923 G(+): *E. coli* ATCC 11775, Yeast: *C. albicans* ATCC 60193 Fungi: *A. niger* ATCC 16404	Broth microdilution method		MBC (mg/mL) *S. aureus*: 7 *E. coli*: 5.6 MFC (mg/mL) *C. albicans*: 12 *A. niger*: 14	[116]

Abbreviations: CC: cytotoxic concentration; EC: effective concentration; HSV: herpes simplex virus; IC: inhibitory concentration; IZ: inhibition zone; MIC: minimum inhibitory concentration; MBC: minimum bactericidal concentration; MFC: minimum fungicidal concentration; MRSA: methycillin-resistant *Staphylococcus aureus*; PI: percentage of inhibition; SI: selectivity index; TI: therapeutic index; VII: viral inhibition index.

**Table 5:** Marine invertebrate lipids or lipid-rich extracts with antimicrobial potential, their origin and extraction method.

Scientific Name	Phylum (Class)	Collection Site	Extracting Solvent(s)/Method	Isolated Lipids or Lipid Classes	Compound Identification Methods	Ref.
*Acanthodendrilla* sp.	Porifera (Demospongiae)	Gokasho Bay, Tokyo, Japan	MeOH. Aqueous residue extracted with Et_2_O and *n*-BuOH. Organic extract fractionated by SiO_2_ (MeOH/CHCl_3_), purified by ODS column and C18 RP HPLC	Steroid sulfates	^1^H and ^13^C NMR	[169]
	Porifera (Demospongiae)	Kundingarengkeke Island, Indonesia	EtOH, acetone and MeOH. Crude extract partitioned between aqueous MeOH and hexane, EtOAc, and BuOH. Hexane extract fractionated on normal-phase SiO_2_ column (*n*-hexane/EtOAc, 7:3, *v*/*v*). Purification by semi-preparative HPLC	Sesterterpenes (Luffariellolide derivatives, Acantholides)	^1^H and ^13^C NMR, ESI-MS, HR-EI-MS	[190]
*Agelas oroides*	Porifera (Demospongiae)	Gökçeada, Northern Aegean Sea, Turkey	MeOH, MeOH/CHCl_3_ (1:1, *v*/*v*) and CHCl_3_. Extract dissolved in MeOH/H_2_O (9:1, *v*/*v*) partitioned against *n*-hexane. *n*-hexane, CH_3_Cl and MeOH extracts fractionated on SiO_2_ (EtOAc (0 → 100%) in hexane). Sephadex LH20 and C18 flash column	FA	^1^H and ^13^C NMR, 1D and 2D NMR, GC-MS, ESI-MS	[162]
*Caminus sphaeroconia*	Porifera (Demospongiae)	Dominica	MeOH extracts chromatographed on Sepahdex LH 20 (MeOH and EtOAc/MeOH/H_2_O 20:5:2). Purification by gradient on SiO_2_ (CH_2_Cl_2_ to CH_2_Cl_2_/MeOH 9:1, *v*/*v*)	Glycolipid (Caminoside)	^1^H and ^13^C NMR, ESI-MS	[147]
		Dominica	MeOH extract purified by Sephadex LH-20 (MeOH). Sephadex LH-20 (EtOAc/MeOH/H_2_O (20:5:2, *v*/*v*))	Glycolipid (Caminoside)	^1^H and ^13^C NMR, ESI-MS	[176]
*Dysidea arenaria*	Porifera (Demospongiae)	Hainan Island, South China Sea, China	CHCl_3_-soluble portion was repartitioned between petroleum ether and 90% MeOH. MeOH extract on flash SiO_2_ column (ether/EtOAc gradient)	Sesquiterpenoid (Sesquiterpenoid hydroquinone)	^1^H and ^13^C-NMR, ESI-MS	[184]
*Dysidea* sp.	Porifera (Demospongiae)	Lakshadweep Islands, Kerala, India	EtOAc and MeOH. EtOAc extract chromatographed on Sephadex LH20 (MeOH/CHCl_3_, 1:1, *v*/*v*), SiO_2_ (2% EtOAc petroleum ether)	Sesterterpenes (Sesterterpene sulfates)	^1^H and ^13^C NMR, HR-FAB-MS	[183]
*Erylus lendenfeldi*	Porifera (Demospongiae)	Gulf of Eilat, Red Sea	MeOH/CHCl_3_, RP on a C18 column (decreasing percentage of H_2_O in MeOH)	Steroidal glycoside (Eryloside)	^1^H and ^13^C NMR, UV, IR	[172]
*Erylus placenta*	Porifera (Demospongiae)	Hachijo Island, Japan	*n*-PrOH/H_2_O (3:1, *v*/*v*). Extracts partitioned between H_2_O and CHCl_3_. H_2_O layer partitioned between *n*-BuOH and H_2_O. BuOH fraction separated by C18 flash (*n*-PrOH/H_2_O (1:9, 3:7, 5:5, and 8:2, *v*/*v*) and CHCl_3_/MeOH/H_2_O(6:4:1, *v*/*v*))	Steroidal glycoside (Sokodosides)	^1^H and ^13^C NMR, GC-FID, UV	[174]
*Euryspongia* sp.	Porifera (Demospongiae)	Light House Reef, Koror, Palau	MeOH. Extracts fractionated by HP20SS column (acetone/H_2_O)	Steroid sulfates (Eurysterols)	^1^H and ^13^C NMR, ESI-MS, UV, IR	[167]
*Fasciospongia* sp.	Porifera (Demospongiae)	Cape Leeuwin, Western Australia	EtOH extract partitioned into *n*-BuOH and H_2_O soluble fractions. *n*-BuOH fraction subsequently defatted by sequential trituration in *n*-hexane and CH_2_Cl_2_ soluble fractions. CH_2_Cl_2_ fractions subjected to SPE or HLPC	Meroterpene (Meroterpene sulfate fascioquinol)	^1^H and ^13^C NMR, HR-ESI-MS, UV	[186]
*Halichondria* sp.	Porifera (Demospongiae)	Unten Port, Okinawa, Japan	Methanolic extract partitioned between H_2_O and EtOAc. EtOAc soluble material subjected to SiO_2_ column (CHCl_3_/MeOH, (95:5, *v*/*v*) and petroleum ether/Et_2_O (9:1, *v*/*v*))	Sesquiterpenoids (Halichonadins)	^1^H and ^13^C NMR, EI-MS, IR	[187]
	Porifera (Demospongiae)		MeOH extract partitioned between H_2_O and EtOAc. EtOAc-soluble material subjected to SiO_2_ column (*n*-hexane/EtOAc, 1:1 → MeOH). MeOH fraction subjected to SiO_2_ column (CHCl_3_/MeOH, 95:5 → 7:3). CHCl_3_/MeOH (7:3) fraction separated on SiO_2_ column (EtOAc/MeOH, 5:1 → MeOH)	Sesquiterpenoid (Halichonadins)	^1^H and ^13^C NMR, ESI-MS, HR-ESI-MS, IR	[188]
*Haliclona simulans*	Porifera (Demospongiae)	Kilkieran Bay, Galway, Ireland	Acetone and MeOH extracts subjected to HP20 chromatography (100% H_2_O → 100% MeOH). Fractionated on flash forward system. SiO_2_ column (100% hexane → 100% EtOAc)	Steroids (24-vinyl-cholest-9-ene-3β,24-diol, 20-methyl-pregn-6-en-3β-ol,5α,8α-epidioxy, 24-methylenecholesterol)	^1^H- and ^13^C NMR, GC-MS	[170]
*Jaspis stellifera*	Porifera (Demospongiae)	Ishigaki Island, Okinawa, Japan	MeOH. EtOAc soluble material subjected to SiO_2_ column (CHCI_3_/MeOH, 9:1 and hexane/EtOAc, 3:7, *v*/*v*). EtOAc-soluble material subjected to SiO_2_ columns and C18 HPLC	Nortriterpenoids (Jaspiferals)	^1^H and ^13^C NMR, EI-MS, UV, IR	[181]
*Luffariella geometrica*	Porifera (Demospongiae)	Great Australian Bight, Australia	CH_2_Cl_2_. Sequential fractionation to obtain pure compounds	Sesterterpenes (Luffarins)	^1^H and ^13^C NMR, EI-MS, UV, IR	[178]
*Luffariella variabilis*	Porifera (Demospongiae)	Western Carolines, Palau	CH₂Cl₂, purified by chromatography	Sesterterpenoids (Manoalide)	^1^H and ^13^C NMR, UV, IR	[180]
		Western Carolines, Palau	CH₂Cl₂, purified by chromatography	Sesterterpenoids (Manoalides)	^1^H and ^13^C NMR, UV, IR	[179]
*Melophlus sarasinorum*	Porifera (Demospongiae)	Makassar, Sulawesi Island, Indonesia	Acetone and MeOH. Extract partitioned between EtOAc and H_2_O. Aqueous extract on HP20 (MeOH, H_2_O). MeOH eluate on C18 (MeOH and H_2_O, gradient elution)	Steroidal glycosides (Sarasinoside)	^1^H and ^13^C NMR, HR-ESI-MS, LC-MS, UV	[173]
*Oceanapia* sp.	Porifera (Demospongiae)	Kamagi Bay, Sada Peninsula, Japan	MeOH extracts partitioned between ether and H_2_O. Organic phase partitioned between *n*-hexane and MeOH/H_2_O (9:1, *v*/*v*). Aqueous MeOH fraction subjected to C18. Purification on SiO_2_ column (CHCl_3_, CHCl_3_/MeOH (9:1), CHCl_3_/MeOH/H_2_O (6:4:1), and MeOH)	Acetylenic acid	^1^H and ^13^C NMR, FAB-MS, UV, IR	[161]
*Paragrantia* cf. *waguensis*	Porifera (Calcarea)	Onna village, Okinawa, Japan	MeOH extract partitioned between H_2_O and EtOAc. EtOAc extract subjected to Sephadex LH20 (CH_2_Cl_2_/MeOH, 1:1, *v*/*v*). Fraction separation on RP HPLC	Acetylenic acid	^1^H and ^13^C NMR, ESI-MS, UV, IR	[163]
*Petrosia weinbergi*	Porifera (Demospongiae)	Acklin Island, Bahamas	MeOH/CHCl_3_ (1:1, *v*/*v*). Aqueous suspension extracted with EtOAc, EtOAc/*n*-BuOH (1:1), and *n*-BuOH. Active extracts fractionated by C18 HPLC	Steroid sulfates (Weinbersterol disulfates)	^1^H and ^13^C NMR, FAB-MS, IR	[166]
*Poecillastra wondoensis, Rhabdastrella wondoensis* (two-sponge association)	Porifera (Demospongiae)	Cheju Island, South Korea	MeOH (70%). Extract partitioned with Et_2_O and H_2_O. Aqueous phase extracted with *n*-BuOH, subjected to C18 flash chromatography and Sephadex LH-20. Purification on C18 HPLC	Steroidal glycosides (Wondosterols)	^1^H and ^13^C NMR, FAB-MS, UV, IR	[175]
*Pseudoceratina purpurea*	Porifera (Demospongiae)	Kaunakakai Harbor, O’ahu island, Hawaii, USA	EtOH and methylene chloride. Combined extracts partitioned (hexane, methylene chloride and BuOH) Isolation: SiO_2_ flash column (hexane). Purification: Sephadex LH-20	Bromotyramine homoserine-derived (Mololipids)	^1^H and ^13^C NMR, HR-FAB-MS, UV, IR	[191]
*Axinyssa digitata*	Porifera (Demospongiae)	Tunisia	Acetone extract partitioned between H_2_O and Et_2_O. Aqueous residue re-extracted with *n*-BuOH and chromatographed on Sephadex LH-20 column (MeOH) and C18 HPLC	Steroid sulfates (Halistanol sulfates)	^1^H and ^13^C NMR, FAB-MS	[165]
*Siliquariaspongia* sp.	Porifera (Demospongiae)	Motualevu reef, Fiji	H_2_O and MeOH/CH_2_Cl_2_ (1:1, *v*/*v*). *n*-BuOH-soluble material from the aqueous extract and CHCl_3_-soluble material from the organic extract chromatographed on Sephadex LH-20 (MeOH/H_2_O, 3:1, *v*/*v*). Purification by RP HPLC	Brominated long-chain acids (Motualevic acids)	^1^H- and ^13^C-NMR, LC-MS, HR-ESI-MS, FT-IR	[164]
*Siphonodictyon coralliphagum*	Porifera (Demospongiae)	Lighthouse Reef and Glover Reef, Belize	EtOH. Aqueous suspension extracted with CH_2_Cl_2_, EtOAc and *n*-BuOH. EtOAc extract fractionated by chromatography and purified on SiO_2_ plates	Phenolic aldehydes (Siphonodictyal)	^1^H and ^13^C NMR, IR, UV	[182]
*Spheciospongia purpurea*	Porifera (Demospongiae)	Weizhou Island, Guangxi Autonomous Region, China	Acetone. Extract resuspended in H_2_O and partitioned with Et_2_O. Et_2_O extract fractionated on SiO_2_ (petroleum ether/acetone, 1:0 → 0:1), Sephadex LH-20 (CH_2_Cl_2_/MeOH, 1:1). Purification by C18 HPLC	Lysophospholipids (PAF(16:0), PAF (16:1 *n*-5), PAF (18:0), PAF (18:1 *n*-7), PAF (18:1 *n*-11), PAF (18:1 *n*-13))	^1^H and ^13^C NMR, IR, ESI-MS, HR-ESI-MS, LC-MS/MS	[146]
Family *Spongiidae*	Porifera (Demospongiae)	Unten Port, Okinawa, Japan	MeOH extract partitioned between EtOAc and H_2_O. H_2_O-soluble portions extracted with *n*-BuOH. EtOAc and *n-*BuOH, soluble materials purified by SiO_2_ columns and C18 HPLC	Sesquiterpenoid (Sesquiterpenoid quinones, Nakijiquinones)	^1^H and ^13^C NMR, FAB-MS, UV, IR	[185]
*Suberites domuncula*	Porifera (Demospongiae)	Rovinj, Croatia	MeOH and CHCl_3_. Combined extracts partitioned between H_2_O and BuOH. Organic layer fractionated by medium-pressure on C18 (linear gradient H_2_O → MeOH → CHCl_3_). Purification by RP HPLC	Lysophospholipids (PAF)	^1^H NMR, FIA-MS, LC-MS/MS	[157]
*Topsentia* sp.	Porifera (Demospongiae)	Chuuk, Federated States of Micronesia	CH_2_Cl_2_/MeOH (1:1, *v*/*v*). Extract fractionated by SPE using C18 cartridges	Steroid sulfates (Eurysterols)	^1^H and ^13^C NMR, HR-ESI-MS, IR, UV	[168]
*Xestospongia* sp.	Porifera (Demospongiae)	Rasch Pass of Madang, Papua New Guinea	Hexane, CH_2_Cl_2_ and EtOAc. Hexane extract subjected to flash column (gradient hexane/EtOAc (95:5, *v*/*v*) → EtOAc)	Brominated FA	^1^H and ^13^C NMR, IR, UV, HR-EI-MS, HR-APCI-MS, HR-FAB-MS	[138]
*Hyas araneus,* *Podopthalmus vigil,* *Lauridromia dehanni, Charybdis helleri, Portunus sanguinolentus,* *Portunus pelagicus*	Arthropoda (Malacostraca)	Vellar Estuary, India	MeOH	Lysoglycerolipids/glycerides FA/esters	^1^H and ^13^C NMR, ESI-MS/MS, FT-IR	[148]
*Meganyctiphanes norvegica*	Arthropoda (Malacostraca)	Straits of Messina, central Mediterranean Sea, Italy	H_2_O/acetone (1:1, *v*/*v*). Supernatant recovered with CH₂Cl₂. Extracts fractionated by semi-preparative RP HPLC-DAD on SB-C8 column	FA (EPA, DHA, ETA)	LC-MS, HPLC-UV-HR-MS	[141]
*Aplidium sp.*	Chordata (Ascidiacea)	Northland, New Zealand	MeOH-CH_2_Cl_2_ extract fractionated with RF C18 flash column (MeOH/H_2_O), Sephadex LH20 (MeOH), semi-preparative C18 HPLC	Meroterpene derivatives (Rossinones)	^1^H and ^13^C NMR, HR-FAB-MS, UV, IR	[189]
*Eunicea succinea*	Cnidaria (Anthozoa)	Mona Island, Puerto Rico,	CHCl_3_/MeOH (1:1, *v*/*v*). Extract fractionated by SiO_2_ column chromatography	FA ((5Z,9Z)-14-methyl-5,9-pentadecadienoic acid)	^1^H and ^13^C NMR, GC-MS, HR-MS, IR	[137]
*Lobophytum crassum*	Cnidaria (Anthozoa)	Rameswaram, India	Aqueous EtOH (95%), MeOH. Extract partitioned with H_2_O and EtOAc. EtOAc extract fractionated on SiO_2_ column (gradient hexane/EtOAc)	Cembranoid diterpene Ceramide	^1^H and ^13^C NMR, FAB-MS, IR	[177]
*Antillogorgia elisabethae*	Cnidaria (Anthozoa)	Bahamas	MeOH. Extract redissolved in EtOH/H_2_O (2:8, *v*/*v*). EtOH/H_2_O extract reextracted with EtOAc, SiO_2_ column (hexane, 0 → 100% EtOAc/hexane, 0 → 100% MeOH/EtOAc). Fractions subjected to RP HPLC (0 → 100% H_2_O/Acetonitrile)	Diterpenes (Elisabethin)	^1^H, ^13^C NMR, HR-EI-MS, UV, IR	[192]
*Sinularia grandilobata,* *Sinularia* sp.	Cnidaria (Anthozoa)	Andaman Islands, India	EtOH. Extract reextracted with EtOAc. Combined extracts fractionated on SiO_2_ column (gradient system hexane/EtOAc (100:0 → 0:100))	Sphingolipids Glycolipids	^1^H and ^13^C NMR, EI-MS	[139]
*Holothuria scabra*	Echinodermata (Echinozoa)	Red Sea, Egypt	EtOH (70%), MeOH, EtOAc and CHCl_3_/MeOH (2:1, *v*/*v*)	Pigments (Carotenoids)	HPLC-UV/VIS, GC-MS	[193]
*Dosidicus gigas*	Mollusca (Cephalopoda)	Hermosillo, Mexico	Acidified MeOH (MeOH/HCl, 99:1, *v*/*v*)	Pigments (Ommochrome)	^1^H and ^13^C NMR, FT-IR,	[144]
*Saccostrea glomerata*	Mollusca (Bivalvia)	Kovalam, Tamilnadu, India	Hexane, EtOAc and MeOH. Purification on SiO_2_ column (hexane/EtOAc and EtOAc/MeOH)	Sterols [Cholesta-5,22-dien-3β-ol, Cholesterol, Ergosta-5,22-dien-3-ol, (3β,22E)-], FA (6-Octadecenoic acid, Octadecanoic acid)	GC-MS, FT-IR	[171]

**Table 6:** Antimicrobial activity of lipids or lipid-rich extracts from marine invertebrates.

Scientific Name	Activity	Tested (Micro)Organisms	Antimicrobial Testing Method/Evaluation	Reference Antimicrobial (Positive Control)	MIC, Diameter of Inhibition Zone (IZ) or Other	Ref.
*Acanthodendrilla* sp.	Antifungal	Yeast: *S. cerevisiae* (A364A, STX338-2C, 14028g, GT160-45C)	Disk diffusion method		IZ (mm): 7–11	[169]
	Antibacterial Antifungal	G(+): *S. aureus, B. subtilis* G(-): *E. coli,* Yeast: *C. albicans* Fungi: *Cladosporium herbarum*	Disk diffusion method		IZ (mm) *S. aureus*: 7–11 *B. subtilis*: 7–12 *E. coli*: 7–12 *C. albicans*: 9–10 *C. herbarum*: 10–20	[190]
*Agelas oroides*	Antibacterial, Antiplasmodial	Acid-fast bacterium: *M. tuberculosis* Parasitic protozoa: *P. falciparum*, *Trypanosoma brucei rhodesiense*, *T. cruzi, L. donovani* G(-): *E. coli*	[^3^H]-hypoxanthine incorporation assay, 96-well microtiter plates, inhibition of enzymatic activity	Artemisinin, Benznidazole, Melarsoprol, Miltefosine, Podophyllotoxin, Triclosan	IC_50_ (µg/mL) Antibacterial *M. tuberculosis:* 9.4–>50 *E. coli*: 0.07–>50 Antiprotozoal: 0.35–>30	[162]
*Caminus sphaeroconia*	Antibacterial	G(+): MRSA*, Enterococcus* (VRE) G(-): *E. coli*	in vitro inhibition		MIC (µg/mL) MRSA: 12 VRE: 12 *E. coli*: > 100	[147]
	Antibacterial	G(+): MRSA*, Enterococcus* (VRE) G(-): *Xanthomonas maltophilia* Plant pathogen: *Pythium ultimum*	Disk diffusion method		MIC (µg/disk) MRSA: 6.3–>100 VRE: 3.1–>100 *X. maltophilia:* 25–>100 *P. ultimum*: 25–>100	[176]
*Dysidea arenaria*	Antiviral	Virus: HIV-1		PFA	IC_50_ (µM) 16.4–239.7	[184]
*Dysidea* sp.	Antibacterial	G(+): *S. aureus, B. subtilis, M. luteus* G(-): *P. vulgaris, S. typhimurium, E. coli*	Broth macrodilution method	Linezolid	MIC (µg/mL) 0.117–>15	[183]
*Erylus lendenfeldi*	Antifungal	Yeast: *C. albicans*			MIC (µg/mL) 15.6	[172]
*Erylus placenta*	Antifungal	G(+): *S. aureus* G(-): *E. coli* Fungus: *Mortierella ramanniana* Yeasts: *S. cerevisiae* (cdc28, act1-1, erg6)	Disk diffusion method		IZ (mm) *M. ramanniana*: 11–12 *S. cerevisiae*: 8–18	[174]
*Euryspongia* sp.	Antifungal	Yeasts: *C. albicans* (ATCC 32354, wild-type) (ATCC 90873, amphotericin B-resistant)	Liquid antifungal assay	Amphotericin B	MIC (µg/mL): 15.6–62.5	[167]
*Fasciospongia* sp.	Antibacterial	G(+): *S. aureus* (ATCC 25923, ATCC 9144), *B. subtilis* (ATCC 6051, ATCC 6633) G(-): *E. coli* (ATCC 11775), *P. aeruginosa* (ATCC 10145) Yeast: *C. albican*s (ATCC 90028)	96-well microtiter plate	Penicillin, Fluconazole	IC_50_ (µM) *S. aureus*: 0.95–2.5 *B. subtilis*: 0.3–7.0	[186]
*Halichondria* sp.	Antibacterial Antifungal	G(+): *M. luteus, B. subtilis* G(-): *E. coli* Yeasts: *C. neoformans,* *C. albicans,* Fungi: *Paecilomyces variotii, A. niger, A. fumigatus*	Broth microdilution method		MIC (µg/mL) *M. luteus*: 0.52 *C. neoformans*: 0.0625 *C. albicans*: 2.09 *P. variotii*: 1.04 *A. niger*: 1.04 *A. fumigatus:* 1.04	[187]
	Antibacterial Antifungal	G(+): *M. luteus* Yeast: *C. neoformans* Fungi: *T. mentagrophytes*			MIC (µg/mL) *M. luteus*: 4 *T. mentagrophytes:* 8–16 *C. neoformans*: 16	[188]
*Haliclona simulans*	Anti-mycobacterial Antitrypanosomal	Acid-fast bacterium: *Mycobacterium marinum* Parasitic trypanosomatida: *T. brucei*	Broth microdilution method	Gentamycin	MIC (µM): *M. marinum*: 156.9–288.8 *T. b. brucei*: 4.58–21.56	[170]
*Jaspis stellifera*	Antibacterial Antifungal	G(+): *Sarcina lutea* Yeast: *C. neoformans* Fungi: *T. mentagrophytes*			MIC (µg/mL) *S. lutea*: 50 *C. neoformans*: 50 *T. mentagrophytes*: 12.5	[181]
*Luffariella geometrica*	Antibacterial	G(+): *S. aureus, Micrococcus* sp*.* Yeast: *S. cerevisiae*	Disk diffusion method		EC (µg/disk) *S. aureus*: 100 *Micrococcus* sp.: 100	[178]
*Luffariella variabilis*	Antibacterial	G(+): *Streptomyces pyogenes, S. aureus*			Active against *S. pyogenes, S. aureus*	[180]
	Antibacterial	G(+): *S. aureus, B. subtilis* G(-): *E. coli, P. aeruginosa* Yeast: *C. albican*s			Active against *S. aureus, B. subtilis*	[179]
*Melophlus sarasinorum*	Antibacterial Antifungal	G(+): *B. subtilis* (DSM2109) G(+): *E. coli* (DSM10290) Yeast: *S. cerevisiae*	Disk diffusion method		IZ (mm) *B. subtilis*: 9 *S. cerevisiae*: 10–13	[173]
*Oceanapia* sp.	Antibacterial Antifungal	G(+): *B. subtilis, S. aureus* G(-): *E. coli, P. aeruginosa* Yeast: *S. cerevisiae, C. albicans* (GT160-45C, cdc5, act1-1, YAT2296c) Fungi: *Penicillium chrysogenum, Mortierella ramanniana*	Disk diffusion method		IZ (mm) *S. cerevisiae*: 6.5–10 *C. albicans*: 8 *E. coli*: 8.5–12.0 *P. aeruginosa:* 8.5–13.0 *B. subtilis*: 11.0 *S. aureus*: 9.5–13.5	[161]
*Paragrantia* cf. *waguensis*	Antibacterial	G(+): *S. aureus* (IAM 12084) G(-): *E. coli* (ATCC 12600)	Broth microdilution method	Rifampicin, Nalidixic acid	MIC (µg/mL) *S. aureus*: 64 *E. coli*: 128	[163]
*Petrosia weinbergi*	Antiviral	Viruses: Feline leukemia virus (FeLV), HIV			EC_50_ (µg/mL) FeLV: 4.0–5.2 HIV: 1.0	[166]
*Poecillastra wondoensis, Rhabdastrella wondoensis* (two-sponge association)	Antibacterial	G(-): *P. aeruginosa*, *E. coli*	Disk diffusion method		Active concentration 10 µg/disk	[175]
*Pseudoceratina purpurea*	Antiviral	Virus: HIV-1			EC_50_ (µM): 52.2	[191]
*Axinyssa digitata*	Antiviral	Viruses: HIV-1, HIV-2			EC_50_ (µg/mL) HIV-1: 3–6 HIV-2: Not referred	[165]
*Siliquariaspongia* sp.	Antibacterial	G(+): *S. aureus,* MRSA	Disk diffusion method, Microbroth dilution		MIC_50_ (µg/mL) *S. aureus*: 1.2–10.9 *S. aureus* (MRSA): 3.9–400	[164]
*Siphonodictyon coralliphagum*	Antibacterial	G(+): *S. aureus,* *B. subtilis*			Active against *S. aureus, B. subtilis*	[182]
*Spheciospongia purpurea*	Antifungal	Yeasts: *C. neoformans* (32609), *C. glabrata* (537) Fungi: *T. rubrum* (Cmccftla), *A. fumigatus* (07544),	Broth dilution	Amphotericin B, Fluconazole, Voriconazole, Ketoconazole	MIC_80_ (µg/mL) *C. neoformans*: 4–32 *C. glabrata*: 8–64 *A. fumigatus*: >64 *T. rubrum*: >64	[146]
Family *Spongiidae*	Antibacterial Antifungal	G(+): *B. subtilis, M. luteus, S. aureus* G(-): *E. coli* Yeasts: *C. albicans, C. neoformans* Fungi: *A. niger*			MIC (µm/mL) *B. subtilis*: 33.3 *E. coli*: >33.3 *M. luteus*: 16.7–33.3 *S. aureus*: 33.3 *C. neoformans*: 8.35 *C. albicans*: 8.35 *A. niger*: 16.7	[185]
*Suberites domuncula*	Antibacterial	Bacterium SB1 (strain isolated from *S. domuncula* with >98.0% similarity to the alpha-*Proteobacterium* (MBIC3368)	Disk diffusion method		IZ (mm): 4.5–6.8	[157]
*Topsentia* sp.	Antifungal	G(+):MRSA, Acid-fast bacterium: *M. intracellulare* Parasitic protozoa: *P. falciparum* (D6 and W2 clones), *L. donovani* Yeasts: *C. albicans, C. glabrata, Candida krusei, S. cerevisiae,* C. *neoformans* Fungus: *A. fumigatus*	Broth microdilution method	Beauvericin	FIC (µM) *C. albicans*: 0.2–1.8 *S. cerevisiae*: 0.08–1.34	[168]
*Xestospongia* sp.	Antibacterial	G(+): MRSA*, S. mutans, S. sobrinus*	Disk diffusion method		IZ (mm) MRSA: 12 *S. mutans:* 17 *S. sobrinus:* 14	[138]
*Hyas araneus, Podopthalmus vigil, Lauridromia dehanni, Charybdis helleri, Portunus sanguinolentus, Portunus pelagicus*	Antibacterial Antifungal	G(+): *S. aureus,* MRSA, *S. pyogenes* G(-): *E. coli*, *P. aeruginosa, S. typhi*, *S. flexneri*, *Klebsiella* sp., *V. cholerae*, *Acinetobacter* sp. Yeasts: *Rhodotorula* sp., *C. albicans*, *C. neoformans* Fungi: *A. fumigatus*, *A. niger*	Disk diffusion method	Penicillin, Ketoconazole	IZ (mm) *S. pyogenes*: 1 *S. typhi*: 1–3 *S. flexneri*: 1–6 *V. cholerae*: 1–4 *Acinetobacter* sp.: 1	[148]
*Meganyctiphanes norvegica*	Antibacterial	G(+): MRSA, MB5393, MSSA, ATCC 29213 G(-): *E. coli* (ATCC 25922)*, K. pneumoniae* (ATCC 700603) Acid-fast bacterium: *M. tuberculosis* (H37Ra ATCC 25177)	Well plate, REMA method	Vancomycin hydrochloride, Aztreonam, Gentamycin sulfate	MIC (µg/mL) MRSA: 80–320 MSSA: 320 *M. tuberculosis*: 320	[141]
*Aplidium sp.*	Antibacterial Antiviral Antifungal	G(+): *B. subtilis* Fungi: *T. mentagrophytes* Virus: HSV-1	Disk diffusion method		IZ (mm) *B. subtilis/T. mentagrophytes:* 3–6 Antiviral activity at 2 μg/disk	[189]
*Eunicea succinea*	Antibacterial	G(+): *S. aureus* (ATCC 25923)*, E. faecalis* (ATCC 29212) G(-): *P. aeruginosa* (ATCC 27853)*, E. coli* (ATCC 25922)	Broth microdilution method		MIC (µmol/mL)/IC_50_ (µg/mL) *S. aureus:* 0.24/36 *E. faecalis*: 0.16/<10	[137]
*Lobophytum crassum*	Antibacterial	G(+): *S. epidermidis, B. subtilis, S. aureus* G(-): *P. aeruginosa*	Disk diffusion method	Ampicillin	IZ (mm) *S. epidermidis*: 9.5–16.5 *B. subtilis:* 8.5–18.0 *S. aureus*: 9.0–19.5 *P. aeruginosa*: 9.0–14.0	[177]
*Antillogorgia elisabethae*	Antibacterial	G(+): *S. pyogenes* (ATCC 19615), *S. aureus* (ATCC 25923), *E. faecalis* (ATCC 19433) G(-): *E. coli* (ATCC 25933), *P. aeruginosa* (ATCC 27853)	Disk diffusion method		MIC (µg/mL)/IZ (mm) *S. pyogenes*: 0.8–1.0/12–17 *S. aureus:* 2.0–2.3/8–11 *E. faecalis:* 3.2–3.8/8–9	[192]
*Sinularia grandilobata,* *Sinularia* sp.	Antibacterial Antifungal	G(+): *B. subtilis* (MTCC 441), *Bacillus pumilus* (NCIM 2327) G(-): *E. coli* (MTCC 443), *P. aeruginosa* (MTCC 1688*)* Yeast: *C. albicans* (MTCC 183) Fungi: *A. niger* (MTCC 1344), *Rhizopus oryzae* (MTCC 1987)	Disk diffusion method		IZ (mm) *B. subtilis*: 11–18 *B. pumilus*: 11–16 *E. coli*: 11–17 *P. aeruginosa*: 11–17 *C. albicans*: 8–17 *A. niger*: 10–16 *R. oryzae*: 10–15	[139]
*Holothuria scabra*	Antibacterial	G(+): *S. aureus* (ATCC 6538), *E. faecalis* G(-): *P. aeruginosa* (ATCC 8739)*, V. damsela, E. coli*	Well-cut diffusion technique		AU *S. aureus*: 1.2–2.8 *E. faecalis*: 1.7–3.2 *P. aeruginosa*: 1.4–1.8 *V. damsela*: 1.6 *E. coli*: 1.2	[193]
*Dosidicus gigas*	Antibacterial Antifungal	G(+): *B. cereus* (CCM 2010)*, Clostridium perfringens* (CCM 4991)*, Listeria monocytogenes* (CCM 4699)*, S. aureus subs. aureus* (CCM 2461) G(-): *Haemophilus influenza (*CCM 4456)*, K. pneumoniae* (CCM 2318)*, S. enterica subs. enterica* (CCM 3807) Yeasts: *C. albicans* (CCM 8186)*, C. glabrata* (CCM 8270), *C. tropicalis* (CCM 8223) Fungi: *Aspergillus clavatus, A. flavus, Aspergillus versicolor, Penicillium chrisogenum, Penicillium griseofulvum, Penicillium expansum*	Disk diffusion method		Inhibition (%) *B. cereus:* 39.4 *C. perfringens:* 45.5 *L. monocytogenes*: 60.7 *S. aureus subs. aureus*: 57.8 *H. influenza*: 54.5 *K. pneumoniae*: 39.4 *S. enterica subs. enterica*: 93.9 C. *albicans*: 66.7 *C. glabrata*: 42.4 *C. tropicalis*: 33.3 *A. clavatus*: 48.4 *A. flavus*: 42.4 *A. versicolor*: 42.4 *P. chrisogenum*: 39.4 *P. griseofulvum*: 42.4 *P. expansum*: 48.5	[144]
*Saccostrea glomerata*	Antibacterial Antifungal	G(+): *S. aureus* G(-): *P. aeruginosa*, *V. harveyi*, *A. hydrophila, P. aeruginosa*, *V. harveyi*, *V. parahaemolyticus* Yeast: *C. albicans* Fungi: *A. niger*, *A. flavus*, *Fusarium* sp. Virus: White spot syndrome virus (WSSV)	Disk diffusion method		IZ (mm) *P. aeruginosa*: 4.1–16.0 *V. harveyi*: 3.8–14.9 *A. hydrophila*: 5.1–14.5 *A. niger*: No activity–High activity *C. albicans:* No activity–High activity *Fusarium* sp: No activity–High activity Antiviral activity: PI < 91.85%	[171]

Abbreviations: AU: activity unit for the clear zone; EC: effective concentration; FIC: fractional inhibitory concentration; IZ: inhibition zone; MIC: minimum inhibitory concentration; PI: percentage inhibition.

## Abbreviations

AMP	Antimicrobial peptide
AMR	Antimicrobial resistance
BuOH	Butanol
CC	Cytotoxic concentration
CH_2_Cl_2_	Dichloromethane
CHCl_3_	Chloroform
CLSI	Clinical and Laboratory Standards Institute (formerly NCCLS)
COSY	Correlation spectroscopy
CRE	Carbapenemase-resistant Enterobacteriaceae
DAG	Diacylglycerol
DGDG	Digalactosyldiacylglycerol
DOX	Dioxygenase
DPA	Docosapentaenoic
EC	Effective concentration
EPA	Eicosapentaenoic acid
ESBL	Extended-spectrum beta-lactamase
ESI-MS	Electrospray ionization-mass spectrometry
ESI-MS/MS	Electrospray ionization-tandem mass spectrometry
ETA	Eicosatetraenoic acid
Et_2_O	Diethyl ether
EtOAc	Ethyl acetate
EtOH	Ethanol
FA	Fatty acid
FAB-MS	Fast atom bombardment-mass spectrometry
FAME	Fatty acid methyl ester
FIA-MS	Flow-injection analysis-mass spectrometry
FT-NMR	Fourier transformed-nuclear magnetic resonance
G(-)	Gram-negative
G(+)	Gram-positive
GC	Gas-phase chromatography
GC-FID	Gas-phase chromatography-flame ionization detector
GC-MS	Gas-phase chromatography-mass spectrometry
GlCer	Glucosylceramide
GRAS	Generally recognized as safe
HMBC	Heteronuclear multiple bond correlation
HPLC	High-performance liquid chromatography
HPLC-DAD	High-performance liquid chromatography-diode-array detector
HPLC-ELSD	High-performance liquid chromatography-evaporative light scattering detector
HPLC-ESI-MS	High-performance liquid chromatography-electrospray ionization-mass spectrometry
HPLC-UV-HRMS	High-performance liquid chromatography-ultraviolet-high-resolution mass spectrometry
HPTLC	High-performance thin-layer chromatography
HR-APCI-MS	High-resolution atmospheric pressure chemical ionization-mass spectrometry
HR-EI-MS	High-resolution-electron ionization-mass spectrometry
HR-FAB-MS	High-resolution fast atom bombardment-mass spectrometry
HRMS	High resolution mass spectrometry
HSQC	Heteronuclear single quantum coherence
HSV	Herpes simplex virus
HSV-1	Herpes simplex virus type 1
HSV-2	Herpes simplex virus type 2
IC_50_	Half maximal inhibitory concentration
IMTA	Integrated multi-trophic aquaculture
IR	Infrared
IZ	Inhibition zone
LC	Liquid chromatography
LC-MS/MS	Liquid chromatography-tandem mass spectrometry
LC_50_	Lethal concentration (ppm) at 50%
LOX	Lipoxygenase
LPC	Lysophosphatidylcholine
MAG	Monoacylglycerol
MBC	Minimum bactericidal concentration
MDR	Multi-drug resistant
MeOH	Methanol
MFC	Minimum fungicidal concentration
MGDG	Monogalactosyldiacylglycerol
MIC	Minimum inhibitory concentration
MIQ	Minimum inhibition quantity
MRSA	Methicillin-resistant *Staphylococcus aureus*
MSSA	Methicillin-sensitive *Staphylococcus aureus*
MUFA	Monounsaturated fatty acid
NCCLS	National Committee for Clinical Laboratory Standards
NMR	Nuclear magnetic resonance
NOESY	Nuclear Overhauser effect spectroscopy
PACT	Photodynamic antimicrobial chemotherapy
PAF	Platelet-activating factor
PC-O	Phosphatidylcholine ether
PE	Phosphatidylethanolamine
PFA	Paraformaldehyde
PI	Phosphatidylinositol
ppm	Parts per million
PrOH	Propanol
PS	Phosphatidylserine
PUFA	Polyunsaturated fatty acid
QSI	Quorum sensing inhibitor
RP	Reversed-phase
SEC	Size exclusion chromatography
SFE	Supercritical fluid extraction
SI	Selectivity index
SiO_2_	Silica gel
SM	Sphingomyelin
SPE	Solid-phase extraction
SQDG	Sulfoquinovosyldiacylglycerol
TI	Therapeutic index
TLC	Thin-layer chromatography
UFA	Unsaturated fatty acid
VII	Viral inhibition index
WHO	World Health Organization
